# easyPARM: Automated, Versatile, and Reliable Force Field Parameters for Metal-Containing Molecules with Unique Labeling of Coordinating Atoms

**DOI:** 10.1021/acs.jctc.4c01272

**Published:** 2025-02-06

**Authors:** Abdelazim M. A. Abdelgawwad, Antonio Francés-Monerris

**Affiliations:** † Institut de Ciència Molecular, 16781 Universitat de València, P.O. Box 22085, València 46071, Spain

## Abstract

The dynamics of metal centers are challenging to describe due to the vast variety of ligands, metals, and coordination spheres, hampering the existence of general databases of transferable force field parameters for classical molecular dynamics simulations. Here, we present easyPARM, a Python-based tool that can calculate force field parameters for a wide range of metal complexes from routine frequency calculations with electronic structure methods. The approach is based on a unique labeling strategy, in which each ligand atom that coordinates the metal receives a unique atom type. This design prevents parameter shortage, labeling duplication, and the necessity to post-process output files, even for very complicated coordination spheres, whose parametrization process remain automatic. The program requires the Cartesian Hessian matrix, the geometry *xyz* file, and the atomic charges to provide reliable force-field parameters extensively benchmarked against density functional theory dynamics in both the gas and condensed phases. The procedure allows the classical description of metal complexes at a low computational cost with an accuracy as good as the quality of the Hessian matrix obtained by quantum chemistry methods. easyPARM v2.00 reads vibrational frequencies and charges in Gaussian (version 09 or 16) or ORCA (version 5 or 6) format and provides refined force-field parameters in Amber format. These can be directly used in Amber and NAMD molecular dynamics engines or converted to other formats. The tool is available free of charge in the GitHub platform (https://github.com/Abdelazim-Abdelgawwad/easyPARM.git).

## Graphical abstract

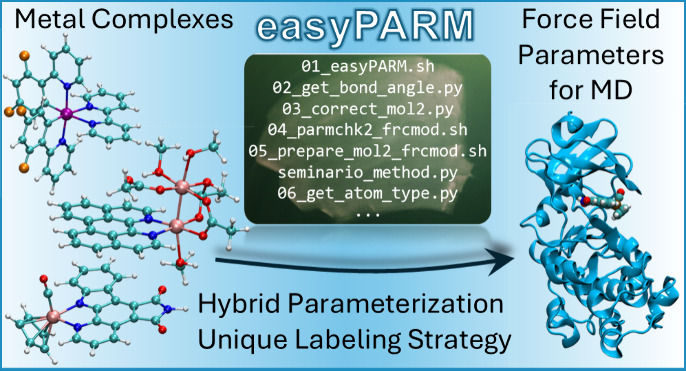

## 1 Introduction

The accuracy of molecular dynamics (MD) simulations strongly relies on the quality of the molecular-mechanics force fields (FFs) used to describe the molecules that compose the system under study.^1^ Traditionally, the development of FF parameters has been strongly oriented to the description of organic molecules with frequent functional groups,^2–4^ or to biological macromolecules such as proteins,^5–9^ nucleic acids,^10–12^ and lipids.^13^ Nevertheless, the presence of metals is ubiquitous not only in biological systems,^14^ but also in photoactive materials,^15,16^ pharmacy and medicine,^17,18^ atmospheric chemistry,^19^ or metal–organic frameworks (MOFs),^20^ among other fields. The parametrization of metal-containing molecules is still not straightforward due to the wide variety of metals and their coordinating spheres composed by organic and/or inorganic ligands around the metal center, hampering the compilation of molecular parameters in ready-to-use general repositories. Thus, widely used tools or Web servers such as Antechamber,^21^ LigParGen,^22^ ATB,^23^ CGenFF,^24,25^ and others^26–30^ usually find parameters absent at best, or cannot be applied at all, obliging users to derive metal-containing FF parameters directly from electronic structure calculations. This has motivated a long-lasting effort of the community in the last decades in developing original protocols and providing shared tools aimed to FF parametrization and refinement of either organic and/or metal containing systems,^31–35^ yielding a wide variety of non-Hessian^36–39^ and Hessian^40–51^ methods and tools.

The growing academic and industrial interest in the design and study of MOFs as innovative materials strongly influences the parametrization and refinement of FFs for metal centers,^49,50,52,53^ which have been successfully applied to describe important MOF properties such as adsorption and diffusion^31,53^ or phonon spectra,^52,54^ among many others.^32^ Non-Hessian methods range from the extension of the universal FF (UFF)^55^ with MOF-specific parametrization (UFF4MOF),^36,37^ the combination of theoretical and experimental data to derive FFs specially tailored for MOFs,^51^ the parametrization of supramolecular structures through QM data (Metallicious),^38^ through the use of periodic density functional theory (DFT) to model mechanical, thermal, and vibrational properties of MOFs.^52^

In the realm of Hessian methods, the Seminario approach^56^ plays a central role due to its relatively simplicity and reasonable accuracy. It consists on the minimization of the differences between QM and molecular-mechanics (MM) Hessian matrices, as first proposed by Dasgupta and Goddard^57^ and Halgren.^58^ As a matter of fact, it has been used in a variety of parametrization tools released in the last years, such as MCPB.py,^47^ VFFDT,^42^ or QUBEKit,^43^ which automates the calculation of bond and angle force constants based on Hessian fittings. Q-Force^44^ integrates QM-derived bonded parameters and atomic charges into classical FFs, striking a balance between accuracy and computational efficiency but facing limitations for organometallic complexes and metal clusters. JOYCE,^46^ released in 2007,^45^ provides protocols for deriving FF parameters directly from QM data, enabling the accurate description of transition-metal complexes dynamics and their properties, as repeatedly demonstrated in previous works.^59–62^ On the other hand, the tool PyConSolv is particularly devoted to parametrize and study the conformational space of molecular systems, including metal-containing complexes.^40^ The reader is referred to the original publications and literature reviews^32^ for further details on available FF parametrization tools.

Whereas many of the mentioned tools are particularly designed to describe MOFs facing lattice periodicities and supramolecular properties, most of the existing FF refinement codes applicable to molecules are not particularly designed for transition-metal complexes. Therefore, the parametrization of whole (usually large) transition-metal complex molecules, although viable, often necessitates careful and extensive manual input and file processing that increases not only the workload but also the required level of expertise of the end user. For instance, Qube-Kit^43^ incorporates virtual sites to model anisotropic electrostatic distributions and focuses on small, relatively rigid organic molecules, while transition-metal complexes are usually of medium to large size and can bear flexible ligands. Parfit^34^ can be computationally intensive for large molecules since it does require geometry sampling, while JOYCE^45,46^ requires the manual parametrization of dihedral angles through individual PES fitting, a process that can be time-demanding when considering large molecules, although it yields high-quality and extremely accurate FF parameters.^59–62^

In this contribution, we present easyPARM, a Python-based tool that automatizes the generation of FF parameters for metal complexes by combining the Seminario method^56^ and the AMBER and GAFF libraries without user supervision. The QM Hessian matrix is used to derive the FF parameters that cannot be retrieved from the transferable FF databases, such as those involving the metal center(s) and, if present, inorganic ligands, while the rest of the molecule is described by retrieving the FF parameters from the AMBER and GAFF libraries. This process is automatized, thereby minimizing workload and human intervention. easyPARM requires the Cartesian Hessian matrix, i.e., the second derivatives of energy with respect to atomic displacements, routinely computed with most electronic structure software packages, to extract the vibrational force constants. Atomic charges are also derived from QM calculations. The tool does not require any connectivity file, atom classification, or atom type list prior parametrization, since these tasks are automatically done by the code. Output files are ready to be used in the system set up and MD simulations without further modification.

To maximize the tool versatility and to ensure the correct description of a great variety of coordination spheres, easyPARM systematically assigns different atom types to each atom that coordinates the metal, regardless of whether these coordinating atoms belong to the same element. We have named this approach the unique labeling strategy (ULS). To the best of our knowledge, ULS is not implemented in any parametrization tool until now. The scheme is illustrated for the six N–Ru–N angles of the octahedral [Ru­(bpy)_3_]^2+^ complex **1** ^63^ shown in Figure 1. In our experience, some parametrization procedures often assign the same atom type to every coordinating atom of the same element (Figure 1a). In this situation, all N atoms bonded to Ru are labeled equally as nb, therefore, all N–Ru–N (nb–Ru–nb) angles have the same equilibrium value and force constant because solely one combination N–Ru–N is defined. However, not all N–Ru–N angles are equivalent, as shown in Figure 1b. Thus, the N_1_–Ru–N_2_ angle, which involves two N atoms of the same bispyridine ligand, must be different than that of N_2_–Ru–N_3_, which consists of two N atoms of different bispyridine units. Therefore, different N atom types must be defined to obtain the correct number of parameter sets; otherwise, the structural dynamics of the complex is compromised. easyPARM and the implemented ULS labels each N atom with a different atom type (six distinct N atoms in the case of **1**), resulting in an extremely flexible and versatile tool that adapts to a huge variety of coordination spheres. Frequent problems encountered in our own experience in managing these atom labels have motivated the development of easyPARM.

**Figure 1:**
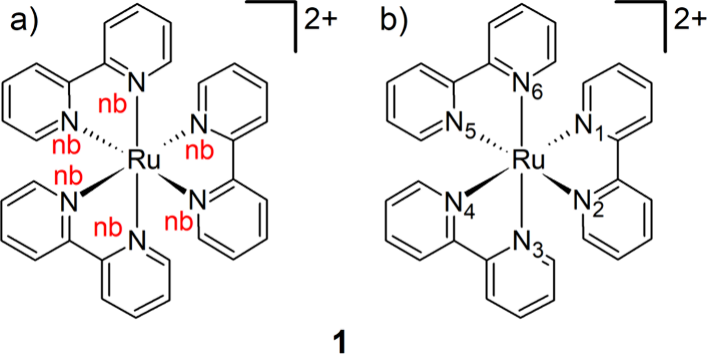
Atom type nomenclature for the atoms that coordinate Ru in [Ru­(bpy)_3_]^2+^ (**1**). (a) Atoms bonded to the metal are described with the same label (nb), and (b) different atom types are assigned to each atom bonded to the metal (ULS).

To validate the accuracy and versatility of easyPARM and the ULS, the metal-containing molecules **1** ^63^ (Figure 1), **2**,^64^ **3**,^65^ **4**,^66^ and **5** ^19^ (Figure 2) were parametrized with this toolkit and simulated in the gas phase. The selected molecules cover a representative variety of metals (Ru, Ir, Re, Pt, and Hg), coordination spheres (**1**, **2**, and **3** are octahedral, **4** is square planar, and **5** is linear), and ligands. The more exotic complexes **6** ^67^ and **7** ^68^ (Figure 2) were modeled in the condensed phase and show that easyPARM automatizes the parametrization process, even for remarkably intricate coordination spheres, providing reliable FF parameters. Relevant bond lengths, angles, and some dihedral angles obtained from the MD simulations using the easyPARM parameters have been benchmarked against ab initio molecular dynamics (AIMD) simulations based on the density functional theory (DFT) method.

**Figure 2:**
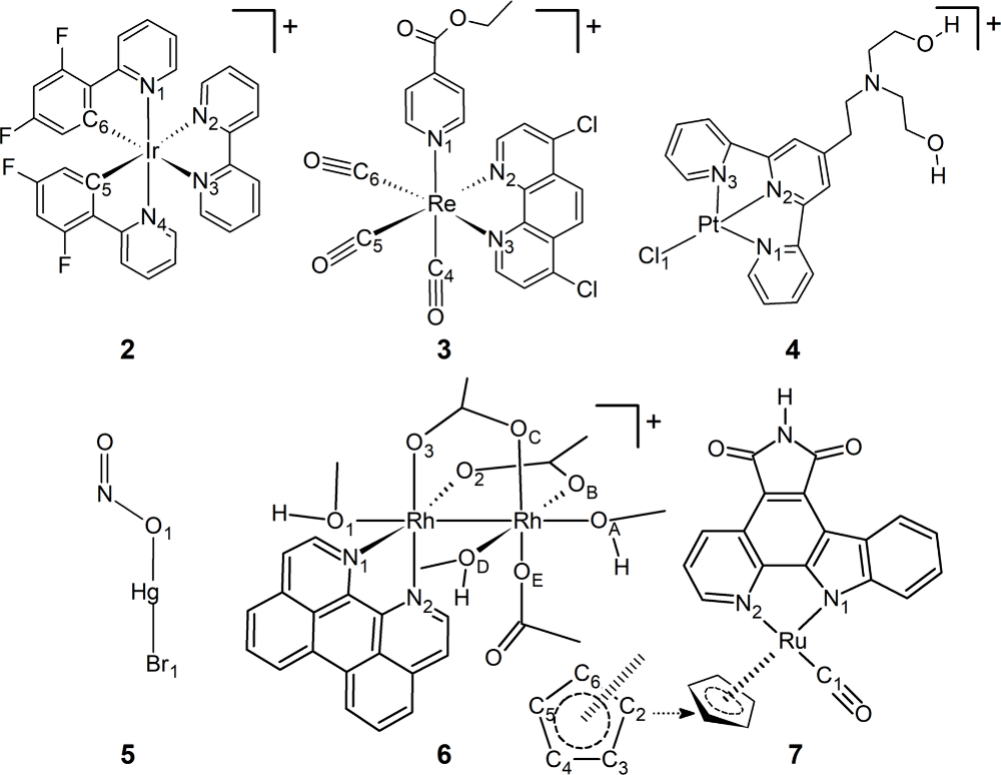
Molecules studied in this work. Some atoms are labeled for analysis.

Results confirm that both easyPARM and AIMD dynamics provide very similar descriptions, demonstrating their effectiveness in preserving critical structural features and confirming the code versatility and reliability. It should be noted that the FF accuracy rests upon the quality of the provided Hessian matrix. Atomic charges can be provided through the usual restricted electrostatic potential (ESP)^69,70^ or CHELPG^71^ methods, although the imposition of charge restraints on one or more specific atoms is also possible in easyPARM through the restrained ESP^72^ fitting. This implementation allows the assignment of a desired atom charge(s) while keeping the charge distribution of the rest of the molecule physically meaningful.

## 2 THEORETICAL METHODOLOGY

In the Amber force field,^73^ the total energy of a system is divided into bonded and nonbonded components:

1 $$U\left(r_{ij}\right)=U_{\mathrm{bonded}}+U_{\mathrm{nonbonded}}$$

where the bonded energy component includes bond stretching, angle bending, and torsional rotation. Bond and angle terms are modeled using harmonic potentials, while torsional energies are described through a Fourier series expansion:

2 $$U_{\mathrm{bonded}}\left(r_{ij}\right)=\underset{\mathrm{all bonds}}{\sum }k_{r}{\left(r_{ij}-r_{ij,\mathrm{eq}}\right)}^{2}+\underset{\mathrm{all angles}}{\sum }k_{\theta }{\left(\theta_{ij}-\theta_{ij,\mathrm{eq}}\right)}^{2}+\underset{\mathrm{all torsions}}{\sum }\underset{n}{\sum }\frac{1}{2}V_{n}\left[1+\cos\left(n\omega -\gamma \right)\right]$$

where *k*_r_, *r*_*ij*_, and *r*_*ij*,eq_ represent the bond force constant, bond length, and equilibrium bond length, respectively, and *k*_θ_, θ_*ij*_, and θ_*ij*,eq_ denote the angle force constant, angle value, and equilibrium angle, respectively. The torsion terms *V*_*n*_, *n*, ω, and γ represent the torsion barrier height, periodicity, torsion angle, and phase offset, respectively. The nonbonded energy includes electrostatic interactions, generally represented by Coulomb’s law, and van der Waals (vdW) interactions modeled using a 12–6 Lennard-Jones (LJ) potential:

$$U_{\mathrm{nonbonded}}\left(r_{ij}\right)=\underset{i,j\neq i}{\sum }\left\{\frac{q_{i}q_{j}}{4\pi \epsilon_{0}r_{ij}}+\varepsilon_{ij}\left[{\left(\frac{r_{ij}}{R_{\min,ij}}\right)}^{12}-2{\left(\frac{r_{ij}}{R_{\min,ij}}\right)}^{6}\right]\right\}$$ 3

where *q*_*i*_ and *q*_*j*_ refer to the partial charges of the particles, *R*_min,*ij*_ represents the distance of minimum energy in the LJ potential, and ϵ_*ij*_ is the well depth in the LJ potential.

Among the several known approaches to incorporating metal ions into force fields,^74–76^ primarily categorized as bonded or nonbonded model approaches, easyPARM adopts the bonded model, which parametrizes only bonds, angles, and electrostatic terms. This strategy follows the approach by Hoops et al.,^77^ which neglects dihedral terms, adopted by other tools^42,47^ and further validated by recent studies.^78–80^ LJ parameters are not parametrized in this model, as most metal ions are embedded within structures where vdW interactions play a lesser role, compared to electrostatic interactions.^81^ Instead, they are retrieved from the UFF,^55^ which contains LJ values for the most common metal ions. The vdW distance (*R*) is modified by halving the UFF value to maintain consistent scaling across both UFF and AMBER force fields.^82,83^

The force constant computations in easyPARM obtained through the Seminario^56^ method are detailed in the following. For a chemical bond between two atoms, the force constant is defined as

4 $$k_{\mathrm{AB}}=\overset{3}{\underset{i=1}{\sum }}{\left({v_{i}}^{\mathrm{AB}}\cdot {û}^{\mathrm{AB}}\right)}^{2}\lambda_{i}^{\mathrm{AB}}$$

where *v*_i_^AB^ is the *i*th eigenvector of the sub-Hessian matrix, extracted from the full Hessian matrix by the code, *û* ^AB^ is the unit vector along the bond axis, and λ_*i*_^AB^ is the *i*th eigenvalue of the sub-Hessian matrix. The calculated bond force constant *k*_AB_ is then converted to kcal/mol/Å^2^ and doubled to adhere to the harmonic approximation. To obtain the angle force constant of any ABC atom triad, sub-Hessians for bonds AB and CB are extracted from the full Hessian matrix. The vectors representing the bonds $\overset{⃗}{\mathrm{AB}}$ and $\overset{⃗}{\mathrm{CB}}$ are calculated as follows:

5 $$\overset{⃗}{\mathrm{AB}}={\mathrm{r⃗}}_{A}-{\mathrm{r⃗}}_{B}$$

6 $$\overset{⃗}{\mathrm{CB}}={\mathrm{r⃗}}_{C}-{\mathrm{r⃗}}_{B}$$

while the normalized unit vectors *û*_AB_ and *û*_CB_ of the bond vectors are

$$\begin{array}{c} {û}_{\mathrm{AB}}=\frac{\overset{⃗}{\mathrm{AB}}}{∥\overset{⃗}{\mathrm{AB}}∥}\\ {û}_{\mathrm{CB}}=\frac{\overset{⃗}{\mathrm{CB}}}{∥\overset{⃗}{\mathrm{CB}}∥} \end{array}$$ 7

The unit normal vector to the plane *û*_N_ containing the angle is

8 $${û}_{N}=\frac{{û}_{\mathrm{CB}}\times {û}_{\mathrm{AB}}}{∥{û}_{\mathrm{CB}}\times {û}_{\mathrm{AB}}∥}$$

The vectors perpendicular to each bond *û*_PA_ and *û*_PC_ are defined as

9 $$\begin{array}{l} {û}_{\mathrm{PA}}={û}_{N}\times {û}_{\mathrm{AB}}\\ {û}_{\mathrm{PC}}={û}_{\mathrm{CB}}\times {û}_{N} \end{array}$$

The sub-Hessians are decomposed by eigenvalue decomposition:

10 $$\begin{array}{l} H_{\mathrm{AB}}=V_{\mathrm{AB}}\Lambda_{\mathrm{AB}}V_{\mathrm{AB}}^{T}\\ H_{\mathrm{CB}}=V_{\mathrm{CB}}\Lambda_{\mathrm{CB}}V_{\mathrm{CB}}^{T} \end{array}$$

Here, Λ_AB_ and Λ_CB_ are diagonal matrices of eigenvalues, and *V*_AB_ and *V*_CB_ are matrices of eigenvectors. The angle force constant *k*_θ_ is calculated using the projections of eigenvectors:

11 $$\frac{1}{k_{\theta }}=\frac{1}{R_{\mathrm{AB}}^{2}\overset{3}{\underset{i=1}{\sum }}\left|{û}_{\mathrm{PA}}\cdot {\mathrm{v̂}}_{i}^{\mathrm{AB}}\right|\lambda_{i}^{\mathrm{AB}}}+\frac{1}{R_{\mathrm{CB}}^{2}\overset{3}{\underset{i=1}{\sum }}\left|{û}_{\mathrm{PC}}\cdot {\mathrm{v̂}}_{i}^{\mathrm{CB}}\right|\lambda_{i}^{\mathrm{CB}}}$$

where *R*_AB_ and *R*_CB_ are the AB and CB bond lengths, respectively. The calculated angle force constant *k*_θ_ is subsequently converted to kcal/(mol rad^2^) and doubled to match the harmonic approximation.

## 3 IMPLEMENTATION

The last version of the code and the manual is available in the GitHub platform (https://github.com/Abdelazim-Abdelgawwad/easyPARM.git). The reader is referred to the program Web site for additional information.

### 3.1 Features

This easyPARM process has the following features:

- easyPARM is specifically designed to parametrize transition-metal complexes. The code expects at least one metal center in the molecule and automatically splits the parameters into two categories: metal-containing and non-metal-containing.
- The unique labeling strategy ensures the correct description of a wide variety of metal complexes, including multimetal centers, preventing parameters shortage and undesired distortions of the metal center.
- Only those parameters involving the metal atom(s) are computed through the Seminario method; the rest are taken from well-known transferable FF databases. This strongly reduces workload and human intervention.
- Bonds and angles that do not contain any metal atom and whose parameters cannot be retrieved from transferable FF databases are also derived using the Seminario method. This allows the FF refinement for transition-metal complexes with purely inorganic ligands.

### 3.2 Prerequisites

easyPARM has been tested only in Linux operating systems. Python 3 or superior and the scipy and periodictable modules are required. Working versions of Gaussian 09 or 16 and Amber are also necessary. Amber18, Amber20, and Amber22 have been tested and deemed as compatible with easyPARM, even though later versions can potentially work as well, provided that the Antechamber module is available.

### 3.3 Input Files

The current easyPARM implementation requires the Cartesian Hessian matrix calculated with the Gaussian^84^ (version 09 or 16) or ORCA^85^ (version 5 or 6) QM codes. This matrix must be generated in either the output file or the checkpoint file (.chk or .fchk). The application also requires two supplementary files, one containing restricted ESP charges (calculated with Gaussian 09/16) or CHELPG charges (calculated with ORCA 5/6), and another with the Cartesian coordinates in *xyz* format. Gaussian 09/16 and ORCA 5/6 input examples to obtain each file can be found in the program manual.

### 3.4 Output Files

Currently, easyPARM provides force field parameters in Amber format.^86^ Standard output files are the parameter modification (.frcmod), library (.lib), Tripos Mol2 (.mol2), and Protein Data Bank (.pdb) files for the nonstandard residue. Note that Amber files can be converted to other formats such as CHARMM or GROMACS with specific scripts distributed with AmberTools (amb2chm_psf_crd.py, amb2gro_top_gro.py). The ParmEd program may enable the conversion to other FF formats such as OpenMM. This list is not exhaustive and other noncommercial tools may be publicly available.

### 3.5 Compatible Metal Complexes

easyPARM automates the parametrization and refinement of FF for metal centers complexing other metals, organic, and/or inorganic ligands. These include usual octahedral cyclometalated species such as **1**, **2**, **3**, or **7**, while bimetallic centers like **6** can also be treated by this tool. Note that the allowed maximum number of linked metals is four. Purely inorganic ligands such as the complex *o*-cobaltabis­(dicarbollide),^87,88^ composed by two [C_2_B_9_H_11_]^2–^ cages η^5^-bonded to a Co­(III) center, can be also parametrized with easyPARM v2.00.

### 3.6 Workflow

The easyPARM package is constituted of a combination of bash and Python scripts and currently has been tested only in Linux systems. The program workflow is outlined in Figure 3 and detailed in the following:

(1) Input file preparation: prior to executing easyPARM, the user must generate the necessary files with QM codes. Gaussian^84^ (version 09 or 16) output or checkpoint (.chk or .fchk) files or ORCA^85^ (version 5 or 6) output file are valid files. The output of the calculation of atomic charges through Gaussian or ORCA is also necessary, and the optimized structure (*xyz* format).
(2) easyPARM start: execute the ./easyPARM.sh interactive bash script. easyPARM will ask a variety of sequential questions to guide the user through the whole parametrization process.
(3) Generation and correction of mol2 files: an initial and most likely incomplete mol2 file is generated from the Gaussian files using the ANTECHAMBER tool from Ambertools by the module 01_easyPARM.sh. Most likely, the connectivity between atoms is not correctly listed in this file since ANTECHAMBER does not recognize metals. The module 02_get_bond_angle.py automatically parses the bond, angle, and dihedral angles in the Cartesian coordinates (*xyz*) file to obtain the correct molecular connectivity, whereas the module 03_correct_mol2.py uses this correct connectivity to amend the previously generated mol2 file, ensuring compatibility with subsequent parametrization steps. The module xyz_to_pdb.py converts an *xyz* file to a PDB file, simplifying the process of generating a MOL2 file for ORCA input.
(4) Creation of frcmod file: The user can choose to retrieve known standard parameters either from the general Amber force field (GAFF)^89^ or from the Amber force field.^90^ These will not be recalculated by easyPARM, which computes only the nonstandard parameters involving metal atoms through the novel ULS. A preliminary frcmod file with the GAFF or AMBER parameters is generated by module 04_parmch2_frcmod.sh. Missing parameters, mostly those involving metal atoms, are flagged for further refinement.
(5) Atom type assignment: The module 05_prepare_mol2_frcmod.py assigns distinct atom types to every atom linked to the metal center. This crucial step allows for differentiation between atoms of the same element that exhibit distinct bonding with the metal center. For more complex structures, such as inorganic compounds, the module 05_prepare_mol2_frcmod_more_atom.py is used to handle atom type assignments.
(6) Missing parameters calculation: the module Seminario_method_GAUSSIAN.py or Seminario_method_ORCA.py applies the Seminario method,^56^ depending on the input format, freshly implemented in a new Python code, to compute the missing parameters identified in the frcmod file. The module reads the Cartesian Hessian matrix as printed in the electronic structure program output.
(7) Frcmod correct file: the modules 06_get_atom_type.py and 07_Seminario_force field.py generate necessary auxiliary files to correct the frcmod file. The module 08_update_force field.py actually replaces the wrong data, yielding the final frcmod file.
(8) File cleaning: easyPARM applies the Seminario method to the whole molecule, and then selectively extracts only the parameters associated with the metal-containing regions or, in general, those not defined in the general databases, to produce the final FF parameters file. This process is automated and not visible to the user. The code generates two files: one containing all parameters calculated using the Seminario method for the entire molecule, and another containing the available parameters retrieved from GAFF or AMBER parameters library. The modules 09_clean_updatedforce field.py and 10_postclean_updatedforce field.py thoroughly review both mol2 and frcmod files searching possible redundant or erroneous information, ensuring that the final files are correct and free from inconsistencies.
(9) Metal nonbonded terms: The nonbonded parameters for the metal atom are obtained from the UFF^55^ using the module 11_retrieve_uffdata.py. The vdW distance *R* is adjusted to half of the original UFF value to maintain consistent scaling between the UFF and AMBER force fields, following previous procedures described in the literature.^42,47,78,91^ Note that LJ parameters are not parametrized, as metal ions are typically situated in environments where electrostatic interactions overcome vdW forces.^92,93^

(10) Library file generation: a library file (.lib) with the full set of force field parameters and molecular topology with the correct connectivity and atomic number is generated by module 12_generate_lib.py. The final file cleaning and preparation are then completed by the module 13_final_clean.py.
(11) Atomic charge restraint: if desired, restrained ESP fitting can be applied to impose a given charge(s) in a certain atom(s). easyPARM utilizes the restrained ESP protocol from AmberTools, and it automatically detects atoms with the same chemical environment, assigning the same charges. New mol2 and library files are then generated with the updated charges by the module 01_easyPARM.sh.

**Figure 3:**
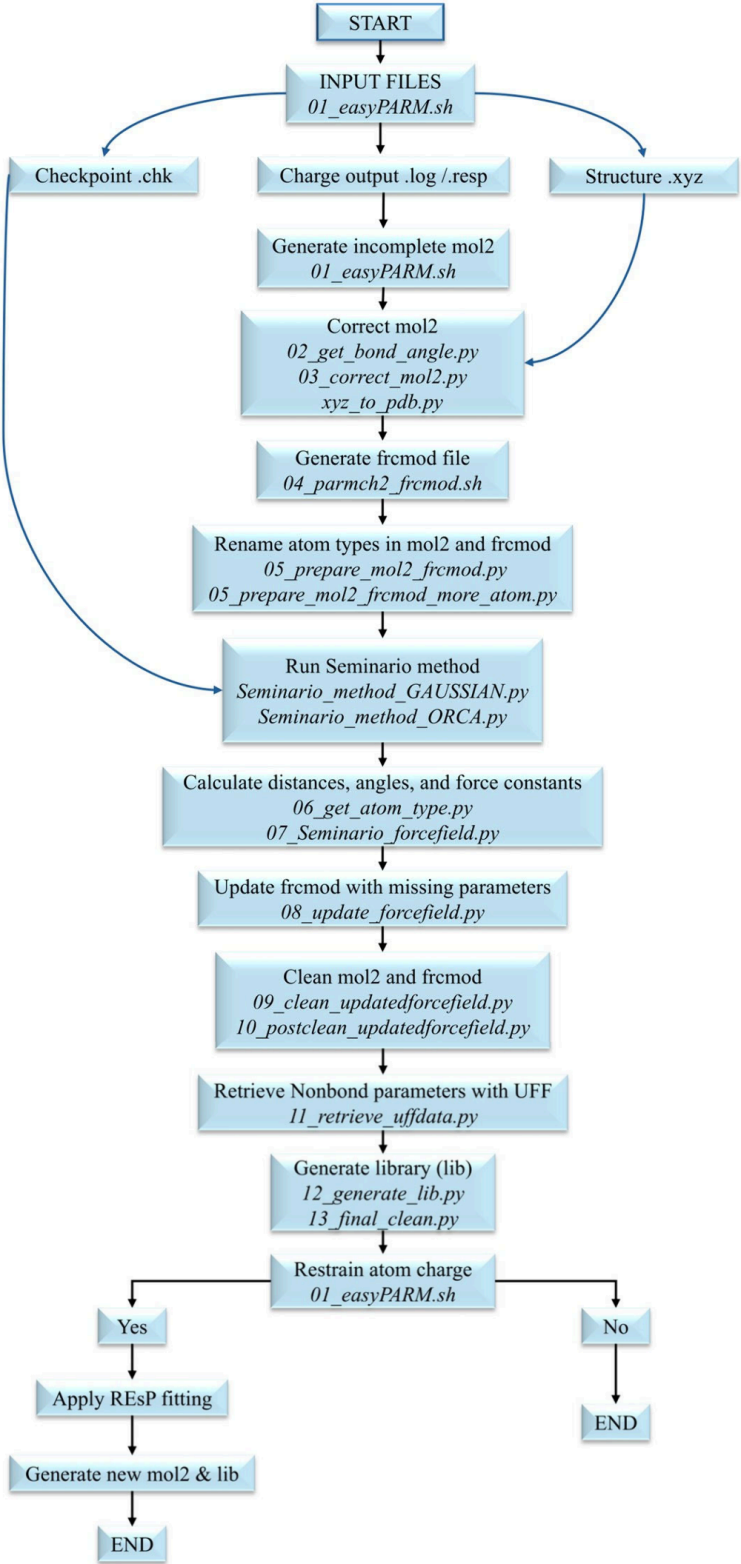
Schematic representation of the easyPARM workflow.

The streamlined, user-friendly process ensures accurate and reproducible force field parametrization for complex metal-containing systems, minimizing the need for human intervention.

## 4 LIMITATIONS

This section informs the reader about limitations in the current implementation of easyPARM (v2.00). Most of these limitations can be addressed or alleviated in future revisions of the software.

- **File format variety.** Currently, the program allows Gaussian^84^ (version 09 and 16) and ORCA^85^ (version 5 or 6) formats for input files, and Amber^86^ format for output files. Although these codes are extremely popular, the tool will benefit from supporting a wider variety of input and output formats. In this regard, noncommercial and publicly available tools to convert easyPARM parameters from Amber to other FF formats may be used to circumvent this limitation at the discretion of the end user. The codes amb2chm_psf_crd.py, amb2gro_top_gro.py, and ParmEd are noncommercially distributed in AmberTools.^86^
- **Number of metals.** Due to technical aspects, the allowed maximum number of linked metals in multimetallic centers is four. Future revisions may expand this number to larger and more-complex multimetal cores.
- **Intrinsic limitations of the Seminario method.** easyPARM is based on a hybrid approach, in which only the parameters involving a metal are derived from QM methods, while the rest of parameters are extracted from general force fields (GAFF or AMBER). This approach minimizes the known shared atoms limitation in the computation of angle force constants, which motivated modifications of the original Seminario method.^35^ Nevertheless, these modifications will be also considered for implementation in this toolkit to further improve the descriptions.

## 5 COMPUTATIONAL DETAILS

### 5.1 Static Quantum-Chemical Calculations

All metal complexes were optimized using DFT, in particular, with the popular B3LYP functional in combination with the 6-31G* basis set for all atoms except the metal, which was described by the quasi-relativistic Stuttgart–Dresden pseudopotential (SDD),^94^ hereafter, referred to as the B3LYP/6-31G*/SDD method. The combination of this functional and the Pople basis set of 6-31G*-type has been successfully used in the optimization of the ground state of different transition-metal complexes, showing reliable structures, compared to experimental observables such as X-ray structures or UV-vis spectra.^95–99^ Following optimization, frequency calculations were performed at the same level of theory to compute the Cartesian Hessian matrixes and to confirm the absence of any negative eigenvalue of the Hessian. Grimme’s D3 dispersion corrections^100^ were included in the geometry optimization and frequency calculations of the largest metal complexes **6** and **7** (Figure 2). Since the impact of this correction, evaluated for molecules **2**, **3**, and **4** (Figure 2), is negligible (see Figures S7–S9, S13–S15, S18, and S19), results shown for complexes **1**–**5** correspond to the parametrization of the Hessian matrix computed without dispersion corrections. All DFT calculations were performed using Gaussian 16 software without any symmetry constraints and the default numerical precision.^84^ While this approach could introduce minor asymmetries in highly symmetric molecules or ligands, users have the flexibility to choose whether to impose symmetry constraints based on their specific needs or even increase the numerical precision during QM optimizations.

### 5.2 Ab Initio Molecular Dynamics

AIMD simulations were carried out using the GPU-accelerated TeraChem software.^101–103^ Each metal complex was simulated for 10 ps with a time step of 1 fs, maintaining the temperature at 300 K with a default thermostat. The B3LYP functional was employed in combination with the 6-31G* basis set for all nonmetal atoms, while metals were treated with the LANL2DZ relativistic pseudopotential (hereafter referred to as the B3LYP/6-31G*/LANL2DZ method).

### 5.3 Gas-Phase Classical Molecular Dynamics

FF parameters for the metal complex were generated with the easyPARM tool by means of the workflow described in the Implementation section. Unless otherwise stated, atomic charges were determined using the restricted ESP protocol^69,70^ in combination with the B3LYP/6-31G*/SDD method. Single-molecule simulations were conducted for each structure with a time step of 1 fs, maintaining the temperature at 300 K for a total simulation time of 10 ps with the Amber 22 software.^86^

### 5.4 Condensed-Matter Classical Molecular Dynamics

FF parameters for the metal complexes were generated with the easyPARM tool. The human Pim-1 kinase was described with the ff14SB force field,^104^ whereas water and counterions were described with the TIP3P parameters. Methanol solvent molecules were treated with the methanol force field as implemented in Amber.^86^ The system (PDB ID: 2BZH) was solvated and neutralized with 12 Na^+^ cations in an octahedral water box with a minimum distance of 10 Å between any protein atom and any box edge. In the case of the simulation of **6** in methanol (see below), the metal complex was introduced in a methanol cubic box in which the distance between any atom of the complex and any box edge was at least 8 Å. After 25 000 and 25 000 steps of minimization with the steepest descent and the conjugated gradient algorithms, respectively, the systems were heated during 200 ps in the NVT ensemble. Then, 10 ps of production runs were simulated in the NPT ensemble, setting the pressure to 1 atm and maintained constant through the Monte Carlo barostat. Temperature (300 K) was kept constant through the Langevin dynamics. Simulations were performed under periodic boundary conditions and utilizing the particle mesh-Ewald (cutoffs of 10.0 and 7.0 Å for **7** and **6**, respectively) and a time step of 1 fs. In the case of the metallodrug:protein system, the production run was extended to 100 ns by using the same settings.

### 5.5 Quantum Mechanics/Molecular Mechanics (QM/MM) Simulations

Metal complexes composed the QM region and were described with the B3LYP/6-31G*/LANL2DZ method, whereas the rest of the system (protein, solvent molecules, and counterions) were treated with the classical force fields described above. The QM-MM cutoff was set to 9.0 and 5.0 Å for systems **7** and **6**, respectively. The total simulation time was 10 ps, with a time step of 1 fs. All QM/MM dynamics were conducted with the Amber/Terachem interface.^86^

### 5.6 Absorption Spectra

To evaluate the capability of the easyPARM parameters in reproducing structural properties such as the absorption spectrum, the UV-vis absorption spectra have been determined for the three representative complexes **1**, **2**, and **3**. Three distinct sets of 25 geometries were generated for each structure: (i) snaphsots extracted from easyPARM MD simulations, (ii) snapshots obtained through AIMD, and (iii) geometries derived from a Wigner distribution via a nuclear ensemble approach (NEA).^105^ For the NEA geometries, Wigner sampling was performed around the ground-state equilibrium geometry using the vibrational normal modes computed with the DFT/B3LYP/6-31G* method, as implemented in the Newton-X 2.0 program.^106^ The absorption spectra for each geometry were calculated by evaluating spin–orbit couplings (SOCs) among the 25 lowest singlet and triplet excited states. These calculations employed the zeroth-order regular approximation (ZORA)^107^ to account for relativistic effects, utilizing the ZORA-Def2-TZVP basis set for all elements except the metal atom, which was treated with the SARC-ZORA-TZVP basis set (with Def2-TZVP/C as auxiliary basis set). Solvent effects (water) were accounted through the conductor-like polarizable continuum model (CPCM),^108^ with default settings in ORCA 5.0.^85^

## 6 Results and Discussion

This section is organized as follows. Simulations with complexes **1**–**5**, conducted in the gas phase, are analyzed first, followed by discussion of the condensed-phase simulations of **6** and **7**. Then, the absorption spectra on complexes **1**, **2**, and **3** are analyzed, finishing with the method to fix atomic charges on certain atom(s). Values of geometrical parameters obtained from the simulations are summarized in Table S1, whereas Figures S1–S25 show the corresponding frequency histograms.

### 6.1 Gas-Phase Simulations

Figure 4 displays the average values and their dispersion (measured with the standard deviation) for selected molecular parameters involving metal atoms. Overall, the agreement between the easyPARM trajectories and the AIMD is excellent, in terms of both average and broadness of the values. Deviations of 0.01–0.06 Å in bond lengths and 1°–2° in bond angles clearly demonstrate the preservation of the structural integrity during the MD trajectories (Table S1 compiles the numerical values represented in Figure 4). The simulation of complex **1** using the labeling scheme shown in Figure 1a with a modified version of easyPARM for that purpose leads to a distorted coordination sphere, as evidenced in Figure S27a and Table S2, whereas the ULS provides a clean octahedral geometry (Figure S27b). easyPARM respects the expected symmetry in the Ru–N distances of **1**, which are, numerically, equivalent (see Table S1). N–Ru–N angles also respect the molecular symmetry. On the other hand, for complex **2**, the Ir–N_1_ average bond distance equals that of the Ir–N_3_ bond, and the same is found for the Ir–N_2_ and Ir–N_3_ bonds and the Ir–C_5_ and Ir–C_6_ bonds, which are also equal in pairs.

**Figure 4:**
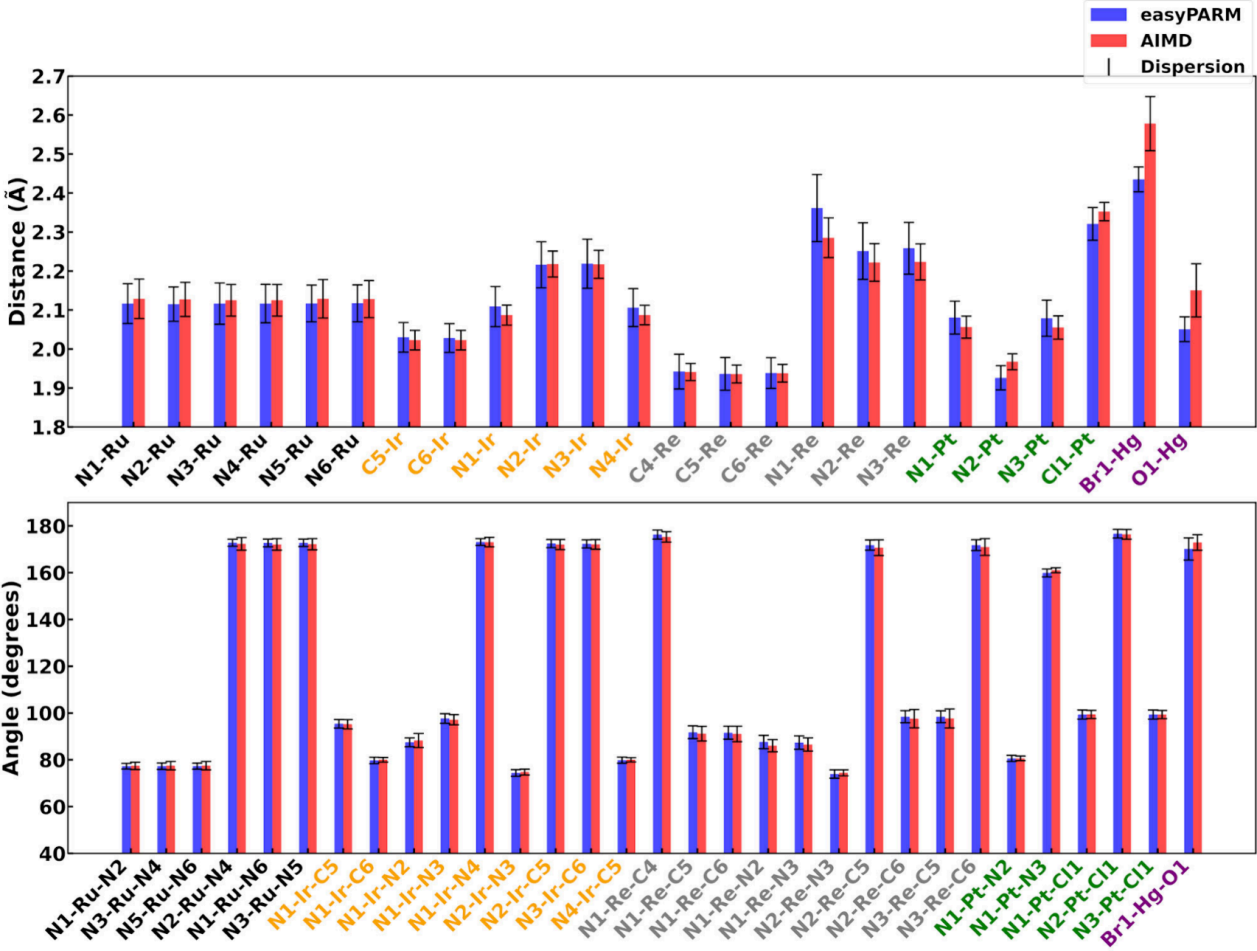
Average (colored thick bars) and dispersion (standard deviation, black thin bars) values for selected molecular parameters involving metal atoms for metal complexes **1** (black), **2** (orange), **3** (gray), **4** (green), and **5** (violet). Each easyPARM and AIMD trajectory was run for 10 ps, extracting the same number of snapshots for the statistical analyses.

easyPARM provides only a slightly wider dispersion of the metal–ligand bond lengths with respect to the AIMD description, as shown by the black bars in the top panel of Figure 4 and in the histograms collected in Figures S1–S25. This systematic difference suggests that the bond force constants calculated via the Seminario method are slightly smaller than that of the DFT dynamics, although the effect of using different MD engines to run the simulations (easyPARM MD are run in Amber 22,^86^ while AIMD are run in Terachem^101–103^ ) could play a role as well, for instance by differences in the temperature control. The comparison reveals small relative errors (≤5.0% for bond distances and ≤8.0% for angles; Table S1 lists the exact values), which is consistent with other parametrization methods,^48^ fully validating the easyPARM toolkit as an automated, versatile, and reliable parametrization method offering an outstanding balance between accuracy and computational cost.

### 6.2 Simulations in Solution

The performance of easyPARM is also benchmarked in this section for the two exotic complex metals **6** and **7** (Figure 2) in different environments (Figure 5). Molecule **6** ^67^ is a Rh-based bimetallic complex that intercalates and coordinates double-stranded DNA. The presence of two metal centers and four types of coordinated ligands makes the parametrization of this complex a challenging task with conventional tools. In this case, the ULS approach facilitates the process by counting all atoms that coordinate a given metal center and considering them as intrinsically different. Oxygen atoms attached to the first Rh center are labeled with numbers from O1 to O3, while the five oxygen atoms coordinated to the second Rh center are labeled with letters, from OA to OE. This is a deliberate generation of distinct labels for each atom, which allows accurate representation of the complex coordination sphere. **6** is studied in a solvent (methanol) octahedral box (Figure 5a) both with classical MD (easyPARM parameters) and QM/MM dynamics at the DFT level, in which the QM partition corresponds to the entire **6** molecule.

**Figure 5:**
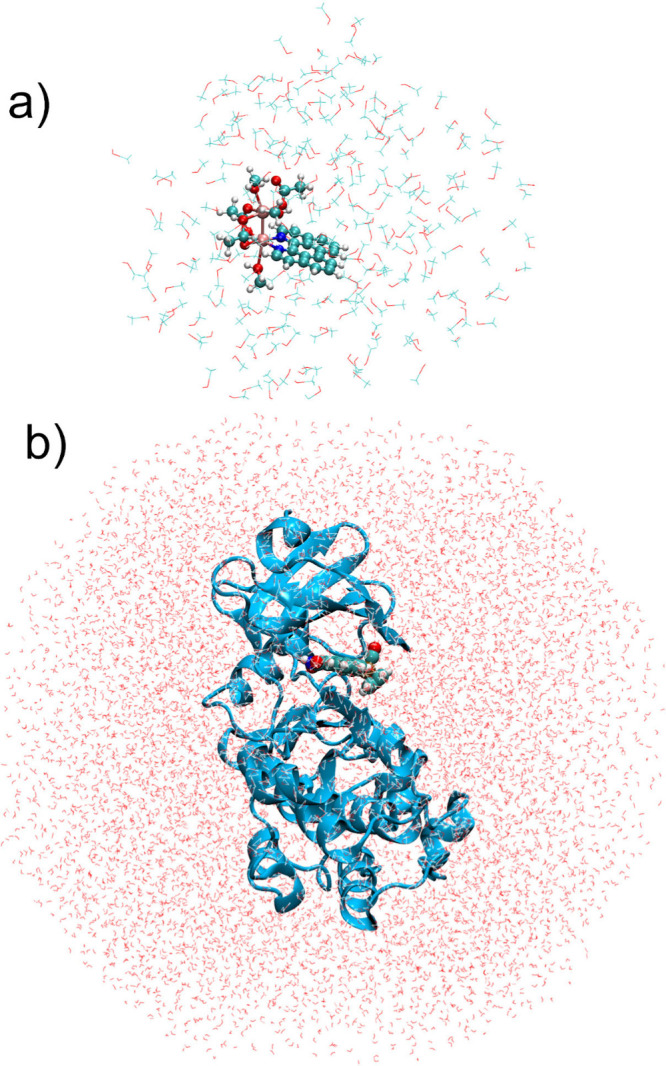
Last snapshot of the (a) complex **6** ^67^ in methanol (10 ps of simulation) and (b) Pim-1 kinase (blue):**7** (balls) complex^68^ after 100 ns dynamics simulated with the easyPARM parameters to describe the metallodrug.

Results show very good agreement between the two methods, in terms of both bond distances and relevant angles (Figure 6). The greatest discrepancies, although acceptable considering the computational cost of the easyPARM parametrization, can be observed in the O_1_–Rh and O_A_–Rh coordination bonds, with an observed relative error between the two methods of ∼17% and 8%, respectively (Table S1). The easyPARM simulation provides almost the same average values for both bonds (2.19 vs 2.17 Å), whereas the O_1_–Rh bond is, on average, longer than the O_A_–Rh (2.64 vs 2.36 Å), despite both bonds being of similar nature (see structure in Figure 2). O_1_ and O_A_ coordination of Rh is relatively weak due to the limited electron donor strength of the oxygen lone pair and its tendency to engage in hydrogen bonding (for example with other methanol molecules from the bulk) rather than forming strong coordination bonds with metals. In the QM/MM simulation, the QM region captures this weakness well, representing the partial bond breaking and forming, leading to more pronounced fluctuations up to longer distances (Figure S21). In addition, the stronger coordinating ligands present in the coordination sphere further weaken the interaction between the methanol ligand and the metal. On the other hand, classical MD simulations do not account for bond breaking/formation, even partial. Instead, the bond fluctuates around a certain distance with a defined force constant (Figure 6). Therefore, easyPARM successfully captures the behavior of the complex, while, in this particular case, the observed shorter O_1_–Rh bond and O_A_–Rh distances are attributed to intrinsic limitations of the classical dynamics based in a force field.

**Figure 6:**
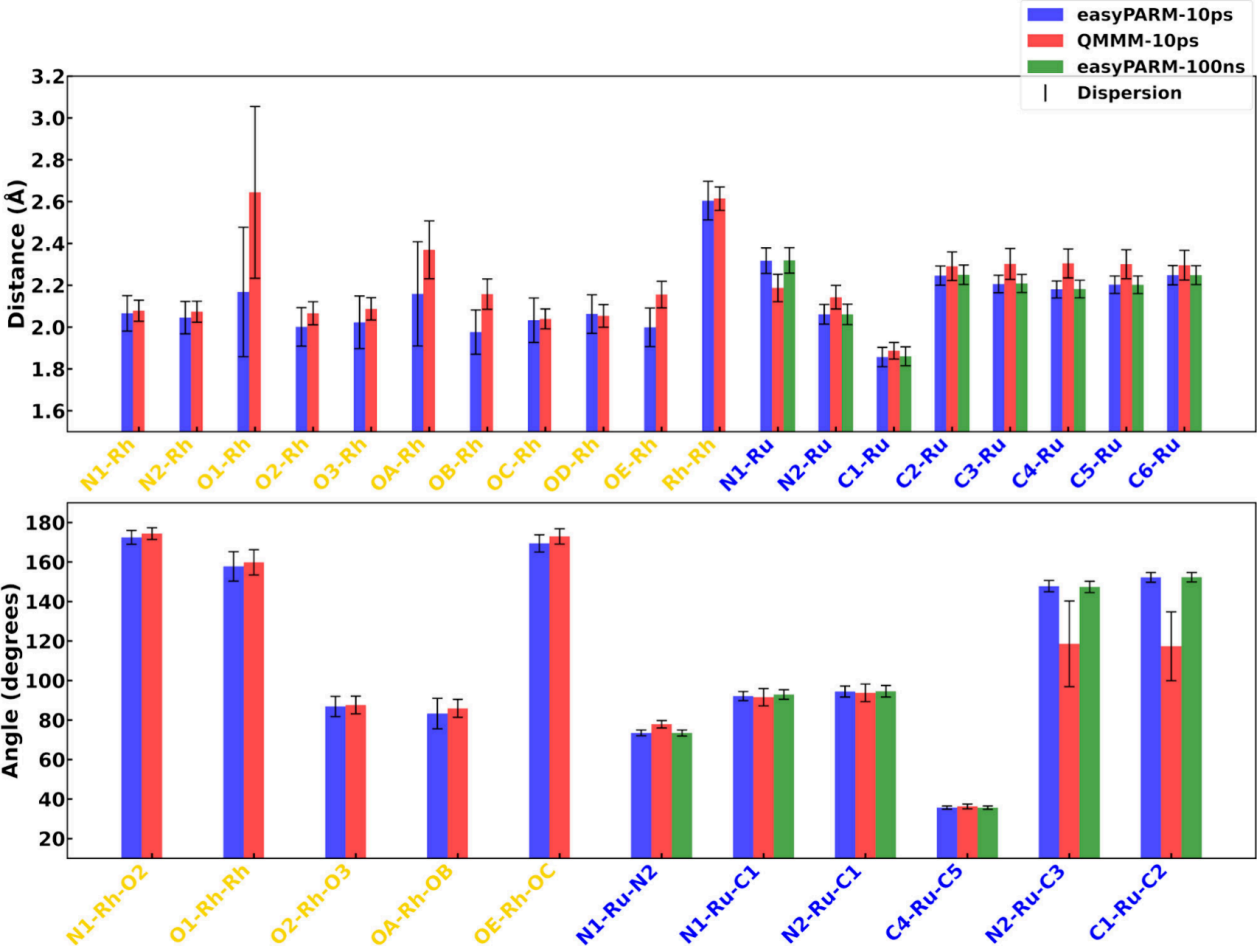
Average (colored thick bars) and dispersion (standard deviation, black thin bars) values for selected molecular parameters involving metal atoms for metal complexes **6** (yellow) and **7** (blue).

The metallodrug **7** ^68^ (Figure 2) has been chosen because the Ru center possesses a coordination sphere rich in variety, coordinating a bidentate aromatic ligand, a carbon monoxide ligand, and cyclopentadienyl anion, greatly complicating the task of parametrization with other available tools. Moreover, **7** is a well-known noncovalent inhibitor of the human Pim-1, kinase due to the large stabilization of the enzyme:drug complex, which allowed the resolution of a crystal structure (PDB ID: 2BZH).^68^ This system offers the opportunity to test whether the easyPARM parameters correctly describe its structural dynamics and capture the protein stabilization described in the literature. Thus, the protein:drug system in water solution (Figure 5b) has been modeled through different methodology. First, two 10 ps trajectories have been propagated, one with the easyPARM parameters and the other one with QM/MM dynamics, in which full complex **7** is included in the QM region. The latter thus serves as reference to benchmark the easyPARM structural dynamics. Second, we have run a 100 ns trajectory with classical MD (easyPARM parameters) to test if the protein stabilization is preserved after significantly longer simulation times.

Analysis of the root-mean-square deviation (RMSD) of the whole system over the 100 ns trajectory indicates that the protein:**7** complex is stable with the easyPARM parameters (Figure S26), whereas the metal–ligand parameters are monitored in Figure 6 and Table S1. Bond distances show a great agreement between the classical and the DFT simulations, with relative errors of ≤6% (Table S1). Largest differences are observed for the angles N_2_–Ru–C_3_ and C_1_–Ru-C_2_, which involve the relative position of the organic (N_2_) and carbon monoxide (C_1_) ligands with respect to the cyclopentadienyl anion (Figure 2). Average values differ by ∼30°–35° and the bending is more flexible in the QM/MM simulations, as provided by the larger standard deviation. Very small differences are observed between the short (10 ps) and long (100 ns) classical MD trajectories using the easyPARM parameters (Figure 6), and therefore, sampling problems can be discarded. The angle discrepancies are thus ascribed to intrinsic inaccuracies of the Seminario method,^56^ which treats angles individually without accounting for the interactions involving shared atoms. This effect can become particularly noticeable when describing an entire complex using the original Seminario method, as the inaccuracies in handling shared atoms can lead to less reliable results. Nevertheless, this limitation is mitigated with the easyPARM hybrid approach, since the Seminario method is used here only to compute the parameters involving the metal atoms, while most of the ligand structures are described with force fields like GAFF^89^ or AMBER,^90^ free from this limitation.

The structure of the Pim-1 kinase:**7** complex (Figure 5b) remains stable after 100 ns of simulation with easyPARM/ff14SB/TIP3P parameters, as shown by the narrow fluctuations of the root-mean-square deviation (RMSD) profile (Figure S26). Figure 7 highlights specific protein–complex interactions of the simulations and the resolved crystal structure. The final distance between the nitrogen and carbon atoms of Gly45 (crystal amino acid numbering) and the oxygen of the carbon monoxide ligand (Figure 7a) is similar to that of the crystal (Figure 7b). The time series of over 100 ns (Figure 7e) shows fluctuations typical of the dynamic simulations. The same analysis applies to the N_2_–Ru-C_3_ angle, whose final value (Figure 7b) and the whole time series (Figure 7f) resembles that of the resolved crystal structure (Figure 7d). Curiously, the QM/MM average value deviates from this value, as discussed in detail above. All in all, these findings validate easyPARM as a reliable tool to study the dynamics of metal-containing molecules in complex environments, offering robust results at low computational cost.

**Figure 7:**
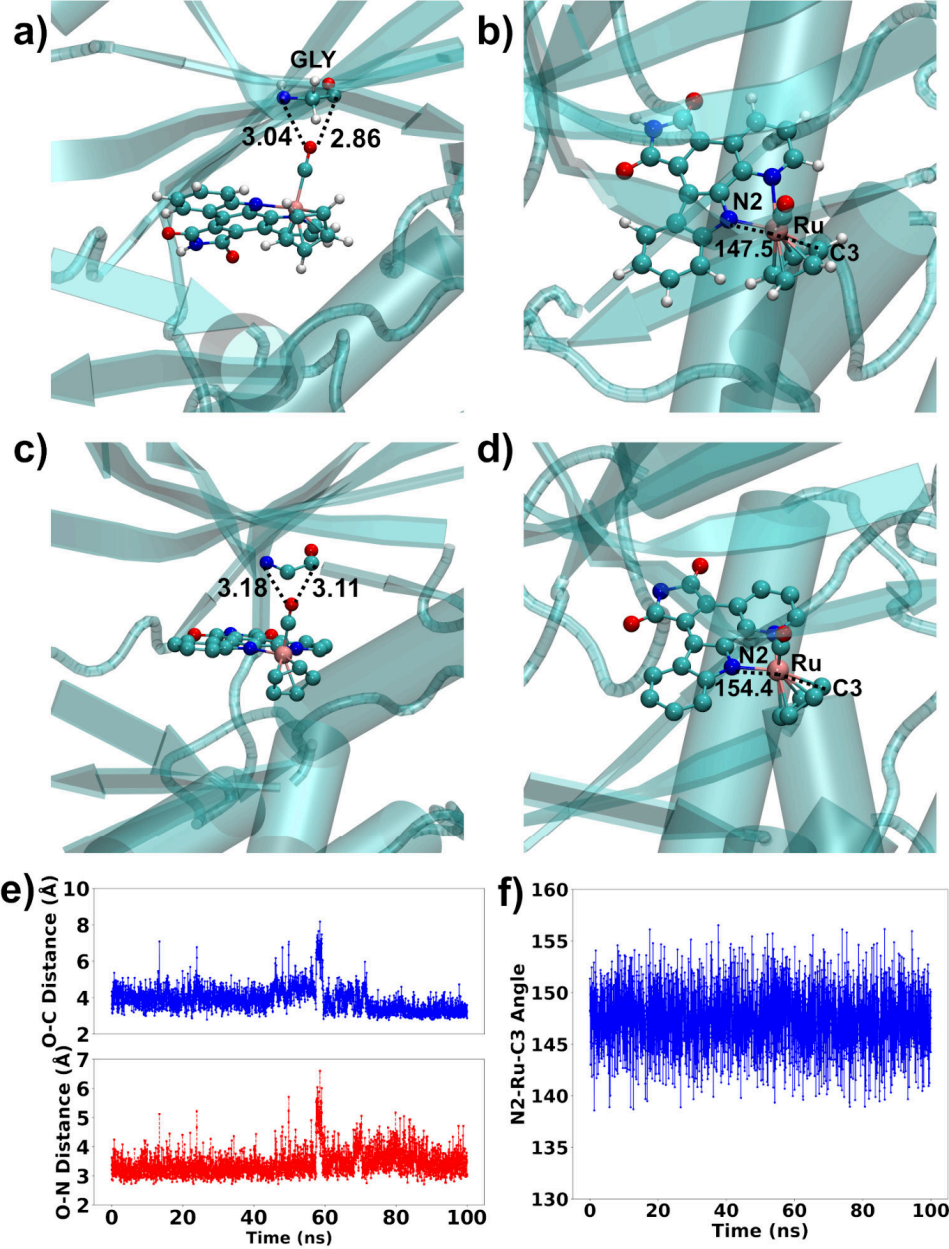
Pim1 kinase:**7** interactions in easyPARM MD simulations (panels (a), (b), (e), and (f)) and in the crystal structure 2BZH^68^ (panels (c) and (d)).

### 6.3 Absorption Spectra

To test the impact of possible geometry discrepancies on other molecular properties that heavily depend on geometrical parameters, Figure 8 displays the absorption spectra for complexes **1**, **2**, and **3** computed on top of easyPARM, AIMD, and nuclear ensemble approach (NEA) sets of geometries. Globally, the strong similarity between the absorption spectra generated with easyPARM and AIMD geometries, respectively, demonstrates that both methods provide very similar structures, thus validating the quality of the easyPARM parameters compared to reference values. Compared with the NEA spectra, both AIMD and easyPARM exhibit a minor blue shift of ∼0.12 eV for the lowest-energy band. This discrepancy is within acceptable limits and is probably due to quantum nuclear sampling (NEA) versus classical sampling (easyPARM, AIMD). For complex **3**, easyPARM geometries show better agreement with the Wigner distribution than those from AIMD, although the differences are again small and within expected deviation values. Finally, Table S3 compares the absorption properties of the Franck–Condon geometries of the minimized geometry using both DFT and easyPARM parameters, showing exactly the same values, again validating the easyPARM parameters, with respect to the DFT reference values.

**Figure 8:**
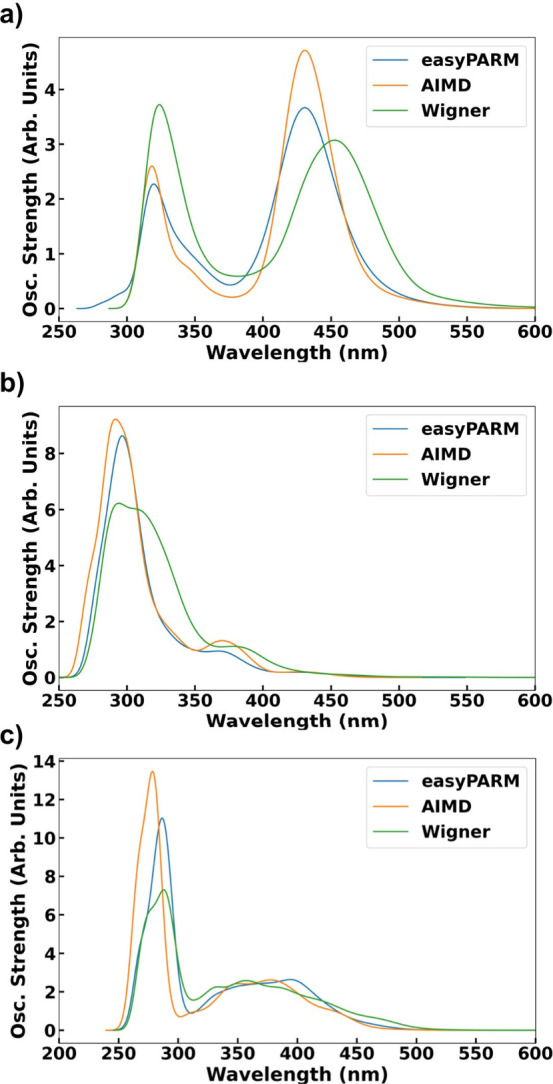
SOC-corrected absorptions spectra for complexes (a) **1**, (b) **2**, and (c) **3**. The convoluted spectra have been obtained by convoluting Gaussian functions centered at each vertical absorption employing a full-width at half-maximum (fwhm) of 0.2 eV.

### 6.4 Charge Restraints on Specific Atoms via the Restrained ESP Procedure

easyPARM allows the use of restraints to impose charge values to one or more atoms of the complex through the restrained ESP method.^72^ Typically, the restricted ESP procedure^69,70^ does not yield accurate atomic charges for metals, often assigning negative values to the metal center, thereby degrading the physical meaning of the charge description. However, the restrained ESP method, as implemented in easyPARM, solves this problem by allowing the imposition of (usual) positive charges to the metal center as appropriate. As an example, this protocol has been applied to impose a +1.45 charge on the Ru atom of complex **1** (Table S4), no physical meaning intended. In any case, appropriate and validated charge values could be sourced from the literature or calculated using alternative methods, such as the Hirshfeld partition scheme.^109,110^ Initial charges fitted via the usual restricted ESP procedure^69,70^ were successfully redistributed by assigning partial charges to atoms at appropriate intensities to replicate the electrostatic field surrounding the molecular surface through the restrained ESP method.^72^ The total charge remains constant, while the Ru restrained ESP charge is 1.45, as desired.

## 7 CONCLUSIONS AND PERSPECTIVES

This contribution presents easyPARM, which is an automated, streamlined, and reliable tool to generate (nontransferable) force-field (FF) parameters for metal-containing molecules based on the unique labeling strategy of all atoms that complex the metal center. By generating as many atom types as necessary, the parametrization procedure based on the Seminario method provides reliable descriptions of the molecular structural dynamics of a wide range of metals and coordination spheres at a very small computational cost. The parametrization strategy is hybrid, combining the quantum-mechanically derived parameters of the metal center with parameters obtained from transferrable force-field databases (AMBER or GAFF), drastically reducing workload and human intervention. The program only requires the Cartesian Hessian matrix computed with Gaussian (version 09 or 16) or ORCA (version 5 or 6) quantum-chemistry software, the geometry file (standard Cartesian coordinates in *xyz* format), and the restricted ESP or CHELPG charges. FF parameters are then returned in ready-to-use Amber format files. The versatility and accuracy of easyPARM have been extensively benchmarked for a variety of metal complexes against DFT simulations both in the gas phase and solution, exhibiting an overall excellent agreement between both descriptions. Atomic charges on metal centers (or any other atoms) can be finely tuned through the restrained electrostatic potential method implemented in easyPARM.

We believe this tool will be of great use to the computational chemistry and molecular modeling community, aiming to describe the dynamic properties of metal-containing complexes, including in silico metallodrug discovery processes. Further expansion of the functions is planned, such as the extension of compatible input and output format files, and the automated parametrization of proteins with metal centers, since this requires the definition of the QM fragment, the use of link atoms, and the proper management of all atom labels. Compatibility expansion with other electronic structure packages, possibly GAMESS,^111^ Q-Chem,^112^ and the open-source PySCF,^113^ is also planned. The last version of the code is publicly available free of cost in the GitHub platform (https://github.com/Abdelazim-Abdelgawwad/easyPARM.git).

## Supplementary Material



## Notes

Average and standard deviation values, histograms of selected bond distances, angles and dihedral angles involving metal atoms, and restricted and restrained ESP atomic charges (PDF)
