# Supplementary material for: easyPARM: Automated, Versatile, and Reliable Force Field Parameters for Metal-Containing Molecules with Unique Labeling of Coordinating Atoms
Source: J Chem Theory Comput. 2025 Feb 6;21(4):1817–30. doi: 10.1021/acs.jctc.4c01272 (PMC12124717; doi:10.1021/acs.jctc.4c01272)
Supplement: Supplementary file 1 [file ct4c01272_si_001.pdf]

SUPPORTING INFORMATION FOR:

easyPARM: Automated, Versatile, and Reliable Force Field Parameters for Metal-Containing Molecules with Unique Labeling of Coordinating Atoms

Abdelazim M. A. Abdelgawwad[1,] and Antonio Francés-Monerris[1,*]*

[1] Institut de Ciència Molecular, Universitat de València, P.O. Box 22085, València 46071, Spain

*A.M.A.A.: abdelazim.abdelgawwad@uv.es; A.F.-M.: antonio.frances@uv.es

Table S1. Average values, standard deviations (SD), and error percentages for selected parameters of all transition metal complexes studied in this work.

Complex	Parameter	easyPARM Average	easyPARM SD	AIMD Average	AIMD SD	Deviation (x_D) (%)[a]
**Bond distances (Å)**						
**1**	N1-Ru	2.1166	0.0511	2.1433	0.0586	-1.25
	N2-Ru	2.1152	0.0441	2.1273	0.0436	-0.57
	N3-Ru	2.1165	0.0529	2.1251	0.0406	-0.40
	N4-Ru	2.1166	0.0493	2.1251	0.0406	-0.40
	N5-Ru	2.1169	0.0470	2.1286	0.0493	-0.55
	N6-Ru	2.1173	0.0475	2.1280	0.0475	-0.50
**2**	C5-Ir	2.0300	0.0381	2.0229	0.0251	0.35
	C6-Ir	2.0302	0.0368	2.0229	0.0251	0.36
	N1-Ir	2.1089	0.0513	2.0870	0.0258	1.05
	N2-Ir	2.2162	0.0590	2.2179	0.0332	-0.08
	N3-Ir	2.2188	0.0629	2.2173	0.0360	0.07
	N4-Ir	2.1063	0.0486	2.0872	0.0250	0.92
**3**	C4-Re	1.9422	0.0446	1.9411	0.0219	0.06
	C5-Re	1.9363	0.0420	1.9358	0.0229	0.03
	C6-Re	1.9385	0.0395	1.9379	0.0227	0.03
	N1-Re	2.3616	0.0861	2.2854	0.0510	3.33
	N2-Re	2.2512	0.0725	2.2221	0.0481	1.31
	N3-Re	2.2584	0.0665	2.2234	0.0463	1.57
**4**	N1-Pt	2.0806	0.0421	2.0562	0.0284	1.19
	N2-Pt	1.9262	0.0311	1.9673	0.0205	-2.09
	N3-Pt	2.0808	0.0463	2.0552	0.0298	1.25
	Cl1-Pt	2.3208	0.0420	2.3524	0.0236	-1.34
**5**	Br1-Hg	2.4352	0.0318	2.5783	0.0695	-5.55
	O1-Hg	2.0507	0.0318	2.1506	0.0682	-4.65
**6**	N1-Rh	2.0658	0.0848	2.0781	0.0504	-0.59
	N2-Rh	2.0606	0.0774	2.0740	0.0502	-0.65
	O1-Rh	2.1681	0.3095	2.6445	0.4107	-18.01
	O2-Rh	2.0012	0.0917	2.0660	0.0553	-3.14
	O3-Rh	2.0229	0.1257	2.0872	0.0532	-3.08
	OA-Rh	2.1591	0.2490	2.3695	0.1384	-8.88
	OB-Rh	1.9764	0.1057	2.1578	0.0723	-8.41
	OC-Rh	2.0329	0.1067	2.0393	0.0477	-0.31
	OD-Rh	2.0627	0.0923	2.0539	0.0542	0.43
	OE-Rh	2.0537	0.0921	2.1558	0.0637	-4.74
	Rh-Rh	2.6046	0.0922	2.6142	0.0561	-0.37
**7**	N1-Ru	2.3174	0.0607	2.1872	0.0655	5.95
	N2-Ru	2.0612	0.0471	2.1430	0.0560	-3.82
	C1-Ru	1.8571	0.0460	1.8865	0.0397	-1.56
	C2-Ru	2.2463	0.0458	2.2911	0.0682	-1.95
	C3-Ru	2.2060	0.0419	2.3020	0.0737	-4.17
	C4-Ru	2.1801	0.0408	2.3046	0.0690	-5.40
	C5-Ru	2.2028	0.0418	2.3007	0.0695	-4.25
	C5-Ru	2.2483	0.0461	2.2958	0.0712	-2.07

Table S1 (cont.). Average values, standard deviations (SD), and error percentages for selected parameters of all transition metal complexes studied in this work.

Complex	Parameter	easyPARM Average	easyPARM SD	AIMD Average	AIMD SD	Deviation (x_D) (%)[a]
			Angles (°)			
1	N1-Ru-N2	77.2544	1.2038	77.4350	1.5603	-0.23
	N3-Ru-N4	77.2838	1.353	77.5022	1.7847	-0.28
	N5-Ru-N6	77.3031	1.2797	77.5022	1.7847	-0.26
	N2-Ru-N5	172.8136	1.4794	172.3404	2.7067	0.27
	N1-Ru-N4	172.7067	1.6422	172.0782	2.4622	0.37
	N3-Ru-N6	172.7639	1.5607	172.1445	2.3850	0.36
2	N1-Ir-C5	95.4068	1.8183	95.1902	1.9939	0.23
	N1-Ir-C6	79.729	1.4027	80.0236	0.9944	-0.37
	N1-Ir-N2	87.4616	1.9189	88.2542	3.0030	-0.90
	N1-Ir-N3	97.6785	2.057	97.1441	2.1706	0.55
	N1-Ir-N4	173.1315	1.4247	173.0628	2.0092	0.04
	N2-Ir-N3	74.3724	1.4525	74.7784	1.2325	-0.54
	N2-Ir-C5	172.4066	1.7467	172.0297	2.1720	0.22
	N3-Ir-C6	172.3077	1.7317	172.0897	2.0364	0.13
	N4-Ir-C5	79.8379	1.3377	80.0077	0.9364	-0.21
3	N1-Re-C4	176.2723	1.9437	175.2985	2.2304	0.56
	N1-Re-C5	91.7874	2.7492	91.1825	3.1432	0.66
	N1-Re-C6	91.5730	2.7971	91.0667	3.3175	0.56
	N1-Re-N2	87.6096	2.8476	86.0428	2.6055	1.82
	N1-Re-N3	87.3872	2.8635	86.5333	2.8266	0.98
	N2-Re-N3	73.9251	1.7934	74.4258	1.2793	-0.67
	N2-Re-C5	171.7974	2.1652	170.6777	3.3437	0.66
	N2-Re-C6	98.4197	2.5613	97.5950	3.9371	0.85
	N3-Re-C5	98.4200	2.5058	97.6817	4.0648	0.76
	N3-Re-C6	171.7686	2.3212	170.9633	3.5593	0.47
4	N1-Pt-N2	80.5927	1.2845	80.5875	0.9861	0.01
	N1-Pt-N3	159.8964	1.6677	161.0178	1.0652	-0.70
	N1-Pt-Cl1	99.3553	1.9638	99.4021	1.7546	-0.05
	N2-Pt-Cl1	176.6661	1.8741	176.3978	2.0983	0.15
	N3-Pt-Cl1	99.3448	1.9575	99.3707	1.7536	-0.03
5	Br1-Hg-O1	170.1222	4.7344	172.8984	3.3478	-1.61
6	N1-Rh-O2	173.9164	3.4854	174.3849	2.9632	-0.27
	O1-Rh-Rh	159.0274	7.4572	159.8521	6.4052	-0.52
	O2-Rh-O3	86.8837	5.1202	87.6252	4.4979	-0.85
	OA-Rh-OB	83.2936	7.7231	85.9450	4.5635	-3.08
	OE-Rh-OC	173.8494	4.3502	172.9472	3.8797	0.52
7	N1-Ru-N2	73.5131	1.4955	77.8847	1.8910	-5.61
	N1-Ru-C1	92.1529	2.3295	91.5775	4.4015	0.63
	N2-Ru-C1	94.4300	2.7936	93.7752	4.4685	0.70
	C4-Ru-C5	35.6868	0.7880	36.2995	1.2150	-1.69
	N2-Ru-C3	147.8039	2.8740	118.5949	21.7111	24.63
	C1-Ru-C2	152.2146	2.4785	117.3736	17.4275	29.68

Table S1 (cont.). Average values, standard deviations (SD), and error percentages for selected parameters of all transition metal complexes studied in this work.

Complex	Parameter	easyPARM Average	easyPARM SD	AIMD Average	AIMD SD	Deviation (x_D) (%)[a]
**Dihedral angles (º)**						
**1**	C-N1-Ru-N6	43.5460	24.0953	43.8468	21.9774	-
	N3-Ru-N5-C	40.7404	20.9951	44.2167	24.0041	-
**2**	N2-Ir-C5-C	-61.7037	22.7526	-66.4415	19.3136	-
	N1-Ir-N4-C	126.8766	41.8748	129.8117	40.6726	-
	C-N1-Ir-N2	125.1350	50.4582	129.8117	40.6726	-
**3**	N1-Re-C5-O	-28.22	92.37	-37.87	82.08	-
	N1-Re-C5-O	-93.8482	9.6713	-95.1747	5.5397	-
**4**	Cl1-Pt-N3-C	-6.2841	174.3460	7.4179	175.6106	-
	Cl1-Pt-N1-C	0.0228	7.2536	0.0633	6.3878	-
	N1-C-C-N2	-0.0753	5.6866	0.0094	5.0321	-
	N2-C-C-N3	0.2668	5.4720	0.0407	4.7451	-
**5**	Br1-Hg-O1-N	2.5759	116.9038	7.4998	111.0195	-

[a] easyPARM average values deviation with respect to AIMD average values. Computed as $x_D = \frac{\bar{x}_{easy} - \bar{x}_{AIMD}}{\bar{x}_{AIMD}} \cdot 100$

Table S2. Average values and standard deviations (SD) for complex **1** using the ULS (easyPARM) and one label approach.

	**ULS**			**One label approach**	
**Parameter**	**Average**	**SD**	**Parameter**	**Average**	**SD**
**Bond distances (Å)**					
N1-Ru	2.1166	0.0511	nb-Ru	2.2238	0.0625
N2-Ru	2.1152	0.0441	nb-Ru	2.2230	0.0699
N3-Ru	2.1165	0.0529	nb-Ru	2.2266	0.0739
N4-Ru	2.1166	0.0493	nb-Ru	2.2291	0.0739
N5-Ru	2.1169	0.0470	nb-Ru	2.2275	0.0746
N6-Ru	2.1173	0.0475	nb-Ru	2.2304	0.0791
**Angles (º)**					
N1-Ru-N2	77.2544	1.2038	nb-Ru-nb	68.4693	2.5750
N3-Ru-N4	77.2838	1.353	nb-Ru-nb	67.4915	2.3182
N5-Ru-N6	77.3031	1.2797	nb-Ru-nb	68.1329	2.2078
N2-Ru-N5	172.8136	1.4794	nb-Ru-nb	130.1435	3.9990
N1-Ru-N4	172.7067	1.6422	nb-Ru-nb	130.5877	3.3303
N3-Ru-N6	172.7639	1.5607	nb-Ru-nb	130.3211	3.9325

Table S3. Vertical excitation energies (E_{VA}) of 25 singlet excited state for complex **1**, **2** and **3** using the minimized structure from easyPARM and the optimized structure from DFT. Numbers between parenthesis refer to oscillator strength.

	1		2		3	
**State**	**E_{VA} (eV) easyPARM**	**E_{VA} (eV) DFT**	**E_{VA} (eV) easyPARM**	**E_{VA} (eV) DFT**	**E_{VA} (eV) easyPARM**	**E_{VA} (eV) DFT**
1	2.59 (0.0003)	2.59 (0.0001)	2.98 (0.0000)	2.98 (0.0000)	3.01 (0.0040)	3.01 (0.0041)
2	2.60 (0.0002)	2.60 (0.0002)	3.36 (0.0901)	3.36 (0.0902)	3.05 (0.1248)	3.05 (0.1244)
3	2.63 (0.0015)	2.64 (0.0015)	3.46 (0.0000)	3.45 (0.0000)	3.18 (0.0065)	3.18 (0.0065)
4	2.72 (0.0001)	2.73 (0.0001)	3.53 (0.0014)	3.53 (0.0014)	3.34 (0.0526)	3.34 (0.0529)
5	2.78 (0.0100)	2.78 (0.0083)	3.58 (0.0713)	3.58 (0.0716)	3.37 (0.0506)	3.37 (0.0509)
6	2.78 (0.0171)	2.78 (0.0125)	3.79 (0.0377)	3.79 (0.0381)	3.45 (0.0003)	3.45 (0.0004)
7	2.86 (0.1398)	2.86 (0.1421)	3.81 (0.0041)	3.81 (0.0041)	3.50 (0.1382)	3.50 (0.1376)
8	2.87 (0.1323)	2.87 (0.1367)	3.88 (0.0027)	3.88 (0.0027)	3.55 (0.0015)	3.55 (0.0015)
9	3.12 (0.0001)	3.11 (0.0001)	3.96 (0.0513)	3.96 (0.0512)	3.65 (0.0203)	3.65 (0.0203)
10	3.42 (0.0085)	3.42 (0.0086)	3.98 (0.0388)	3.98 (0.0373)	3.80 (0.0084)	3.79 (0.0083)
11	3.45 (0.0001)	3.44 (0.0002)	4.00 (0.0923)	4.00 (0.0943)	3.98 (0.0435)	3.98 (0.0456)
12	3.56 (0.0008)	3.54 (0.0001)	4.06 (0.0254)	4.06 (0.0258)	4.06 (0.0001)	4.06 (0.0001)
13	3.56 (0.0146)	3.56 (0.0146)	4.13 (0.0415)	4.13 (0.0417)	4.20 (0.0229)	4.20 (0.0227)
14	3.56 (0.0142)	3.56 (0.0148)	4.15 (0.0133)	4.15 (0.0038)	4.27 (0.0008)	4.27 (0.0009)
15	3.72 (0.0005)	3.72 (0.0010)	4.15 (0.0183)	4.15 (0.0276)	4.41 (0.1149)	4.41 (0.1152)
16	3.76 (0.0010)	3.76 (0.0009)	4.17 (0.2919)	4.17 (0.2949)	4.42 (0.3165)	4.42 (0.3148)
17	3.78 (0.0017)	3.76 (0.0015)	4.20 (0.0010)	4.21 (0.0010)	4.46 (0.0187)	4.46 (0.0186)
18	3.82 (0.0004)	3.83 (0.0000)	4.25 (0.2556)	4.25 (0.2548)	4.48 (0.0288)	4.48 (0.0291)
19	3.87 (0.0006)	3.86 (0.0011)	4.25 (0.0031)	4.25 (0.0023)	4.54 (0.0157)	4.54 (0.0165)
20	3.87 (0.0007)	3.87 (0.0006)	4.32 (0.0200)	4.32 (0.0201)	4.57 (0.0016)	4.57 (0.0017)
21	3.88 (0.0001)	3.88 (0.0001)	4.34 (0.0239)	4.34 (0.0246)	4.60 (0.0627)	4.60 (0.0620)
22	3.91 (0.0469)	3.91 (0.0491)	4.40 (0.0088)	4.40 (0.0086)	4.63 (0.2821)	4.63 (0.2785)
23	3.91 (0.0372)	3.91 (0.0414)	4.44 (0.1901)	4.44 (0.1904)	4.69 (0.1218)	4.69 (0.1261)
24	3.92 (0.0316)	3.92 (0.0296)	4.49 (0.0001)	4.49 (0.0002)	4.70 (0.0021)	4.70 (0.0013)
25	3.92 (0.0466)	3.92 (0.0412)	4.56 (0.0003)	4.56 (0.0003)	4.71 (0.0002)	4.71 (0.0005)

Table S4. RESP and REsP charges for complex **1** obtained with different protocols implemented in easyPARM.

Atom	Restricted electrostatic potential	Restrained electrostatic potential
C1	-0.030388	-0.026678
C2	-0.134232	-0.029800
C3	-0.115082	-0.179809
N1	0.300399	-0.233025
C4	0.051227	0.098063
C5	-0.169043	-0.134201
C6	0.051227	0.098063
N2	0.300399	-0.233025
Ru1	-1.390807	1.450000
N3	0.300399	-0.233025
C7	-0.115082	-0.179809
C8	-0.134232	-0.029800
C9	-0.030388	-0.026678
C10	-0.169043	-0.134201
C11	0.051227	0.098063
C12	0.051227	0.098063
N4	0.300399	-0.233025
C13	-0.115082	-0.179809
C14	-0.134232	-0.029800
C15	-0.030388	-0.026678
C16	-0.169043	-0.134201
N5	0.300399	-0.233025
C17	0.051227	0.098063
C18	0.051227	0.098063
C19	-0.169043	-0.134201
C20	-0.030388	-0.026678
C21	-0.134232	-0.029800
C22	-0.115082	-0.179809
N6	0.300399	-0.233025
C23	-0.169043	-0.134201
C24	-0.030388	-0.026678
C25	-0.134232	-0.029800
C26	-0.115082	-0.179809
C27	-0.115082	-0.179809
C28	-0.134232	-0.029800
C29	-0.030388	-0.026678
C30	-0.169043	-0.134201
H1	0.163031	0.146589
H2	0.174345	0.151970
H3	0.157500	0.146589
H4	0.167378	0.151970
H5	0.157500	0.146589
H6	0.174345	0.151970
H7	0.163031	0.146589
H8	0.167378	0.151970
H9	0.157500	0.146589
H10	0.174345	0.151970
H11	0.163031	0.146589
H12	0.167378	0.151970
H13	0.167378	0.151970
H14	0.163031	0.146589
H15	0.174345	0.151970
H16	0.157500	0.146589
H17	0.167378	0.151970
H18	0.163031	0.146589
H19	0.174345	0.151970
H20	0.157500	0.146589
H21	0.157500	0.146589
H22	0.174345	0.151970
H23	0.163031	0.146589
H24	0.167378	0.151970

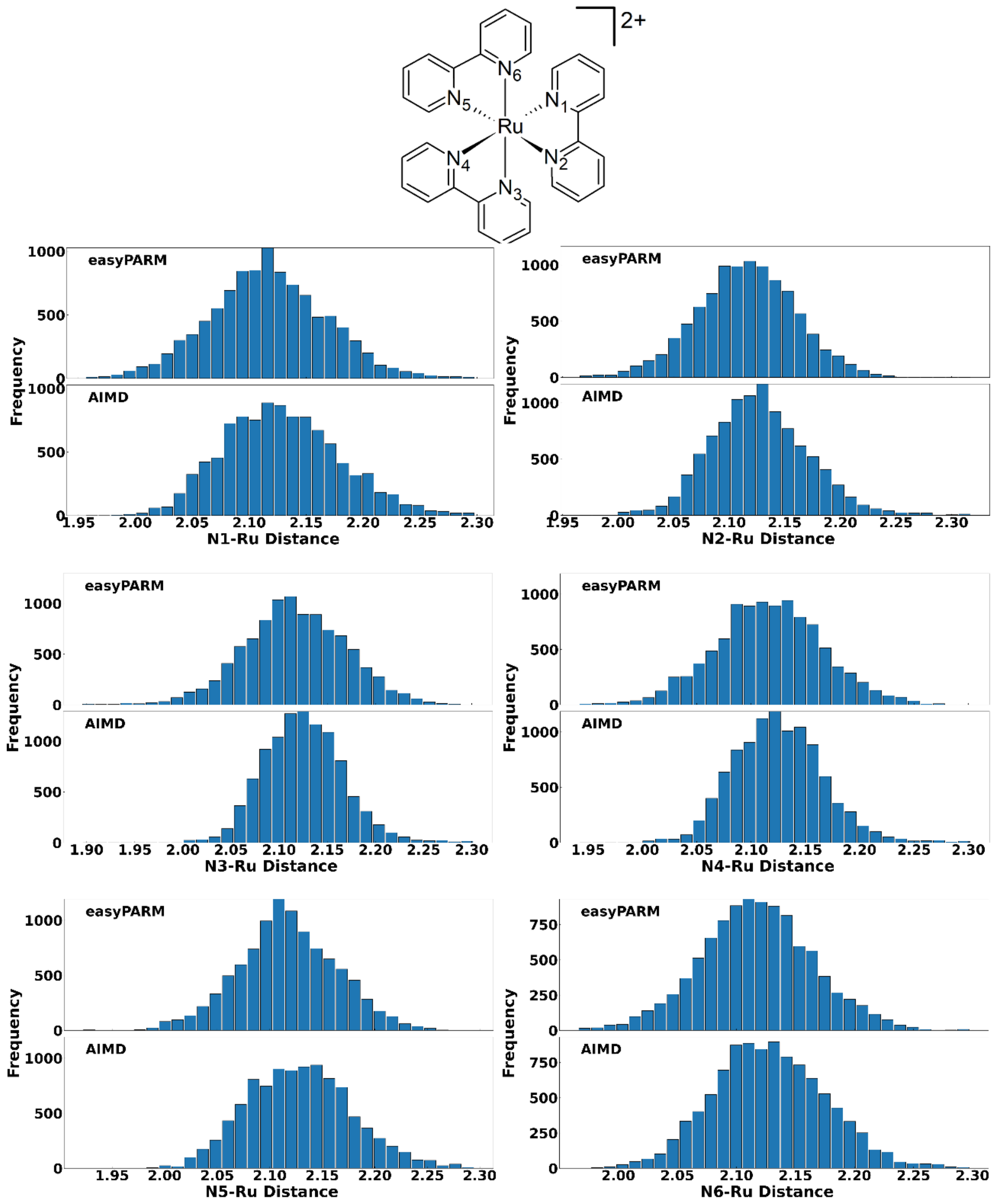

Figure S1. Histograms of selected parameters involving the metal center for Structure **1**, comparing easyPARM (MD) and *ab initio* molecular dynamics (AIMD) over 10 ps.

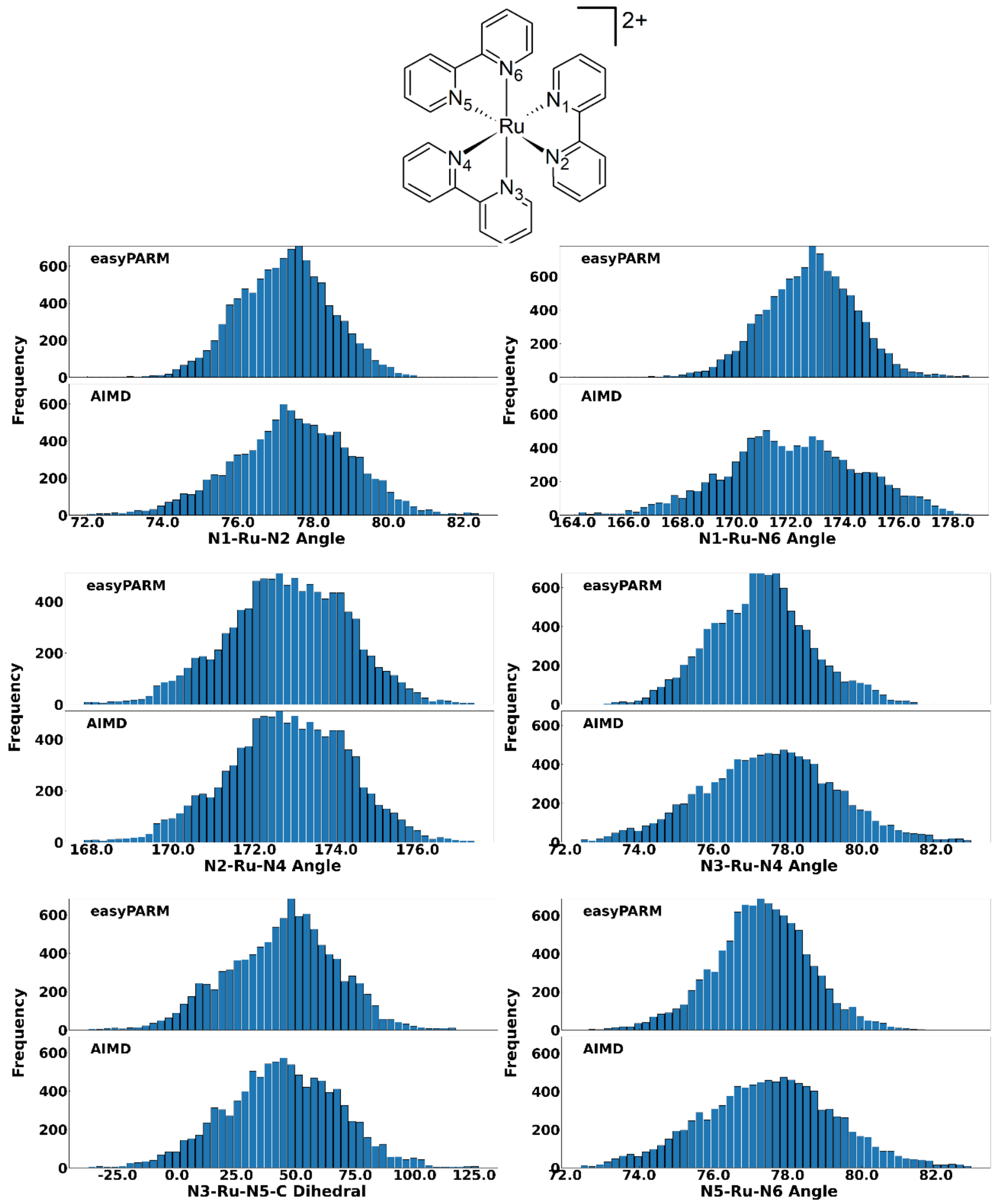

Figure S2. Histograms of selected parameters involving the metal center for Structure **1**, comparing easyPARM (MD) and *ab initio* molecular dynamics (AIMD) over 10 ps.

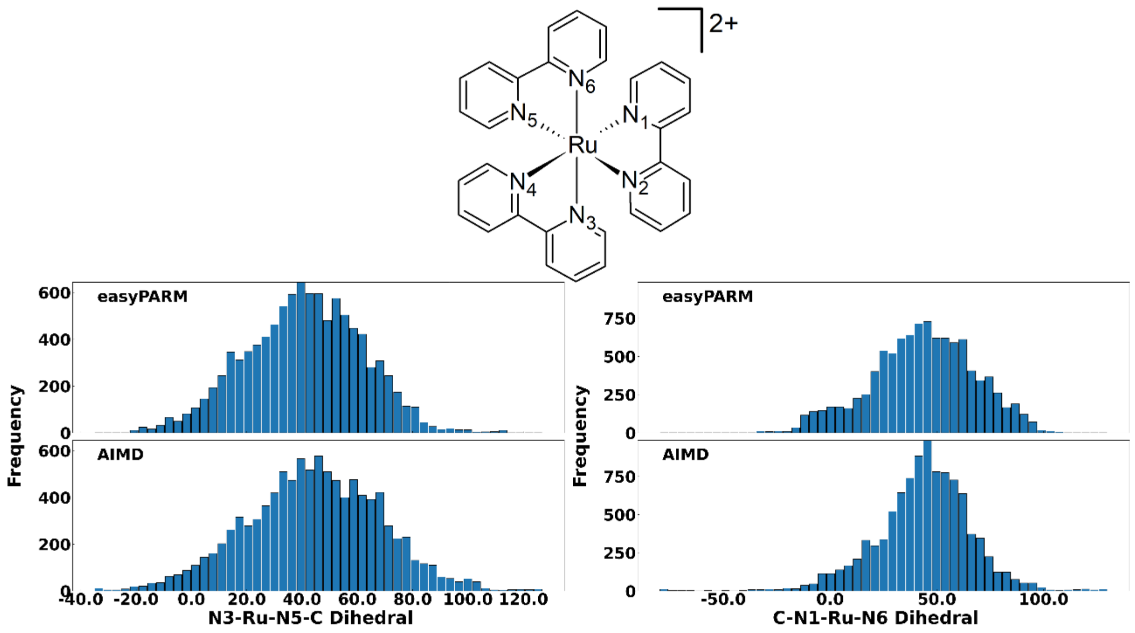

Figure S3. Histograms of selected parameters involving the metal center for Structure **1**, comparing easyPARM (MD) and *ab initio* molecular dynamics (AIMD) over 10 ps.

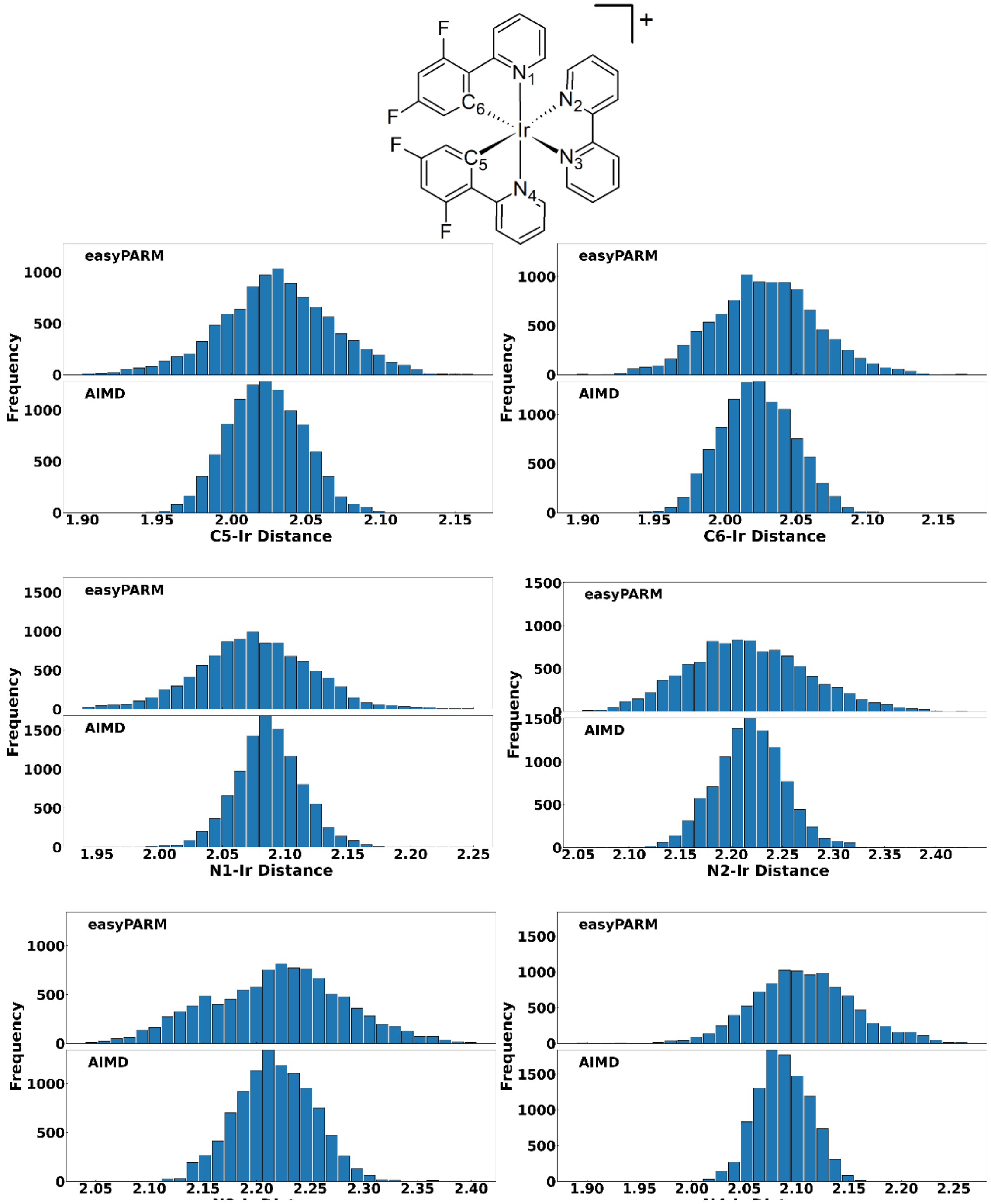

Figure S4. Histograms of selected parameters involving the metal center for Structure **2**, comparing easyPARM (MD) and *ab initio* molecular dynamics (AIMD) over 10 ps.

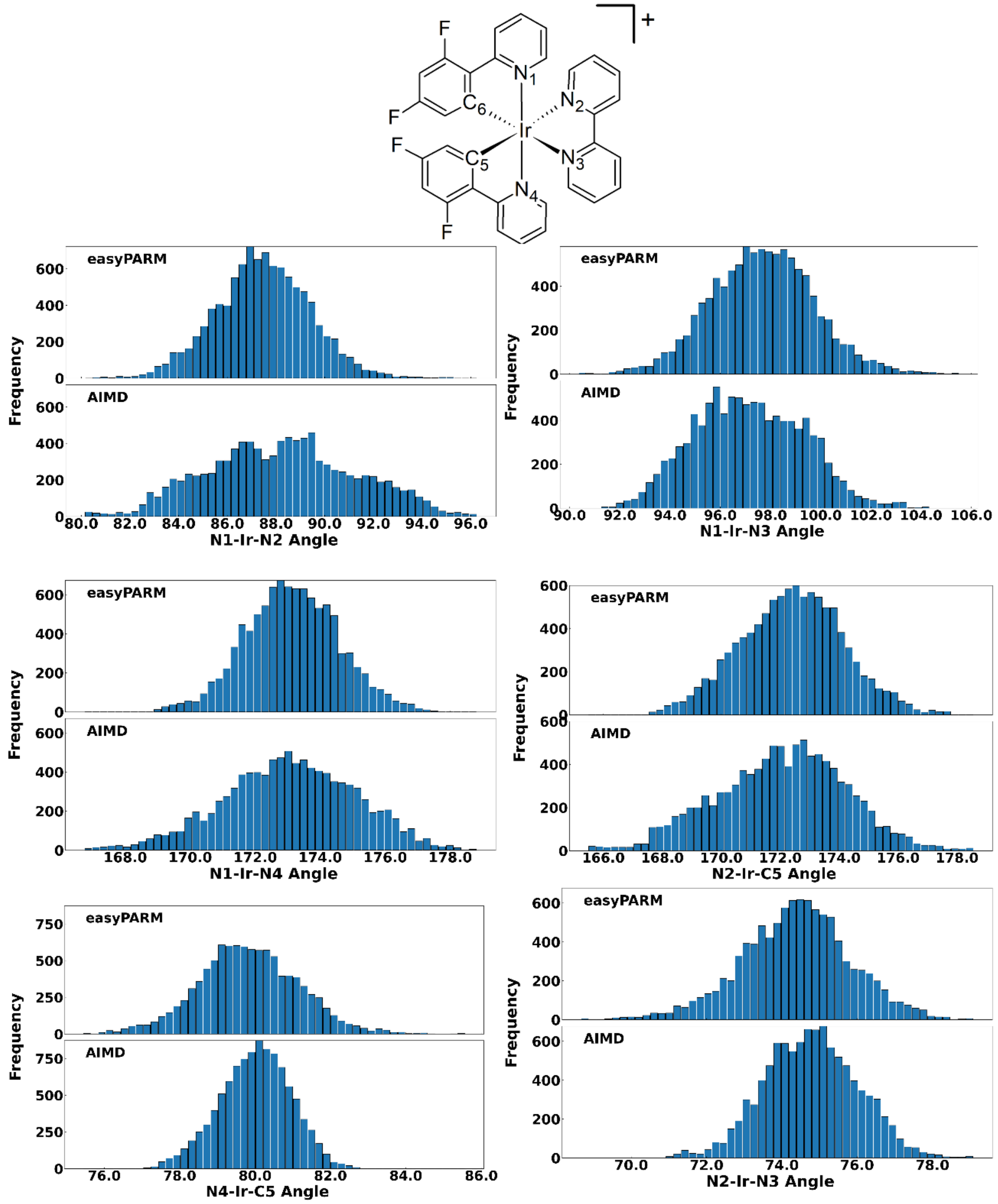

Figure S5. Histograms of selected parameters involving the metal center for Structure **2**, comparing easyPARM (MD) and *ab initio* molecular dynamics (AIMD) over 10 ps.

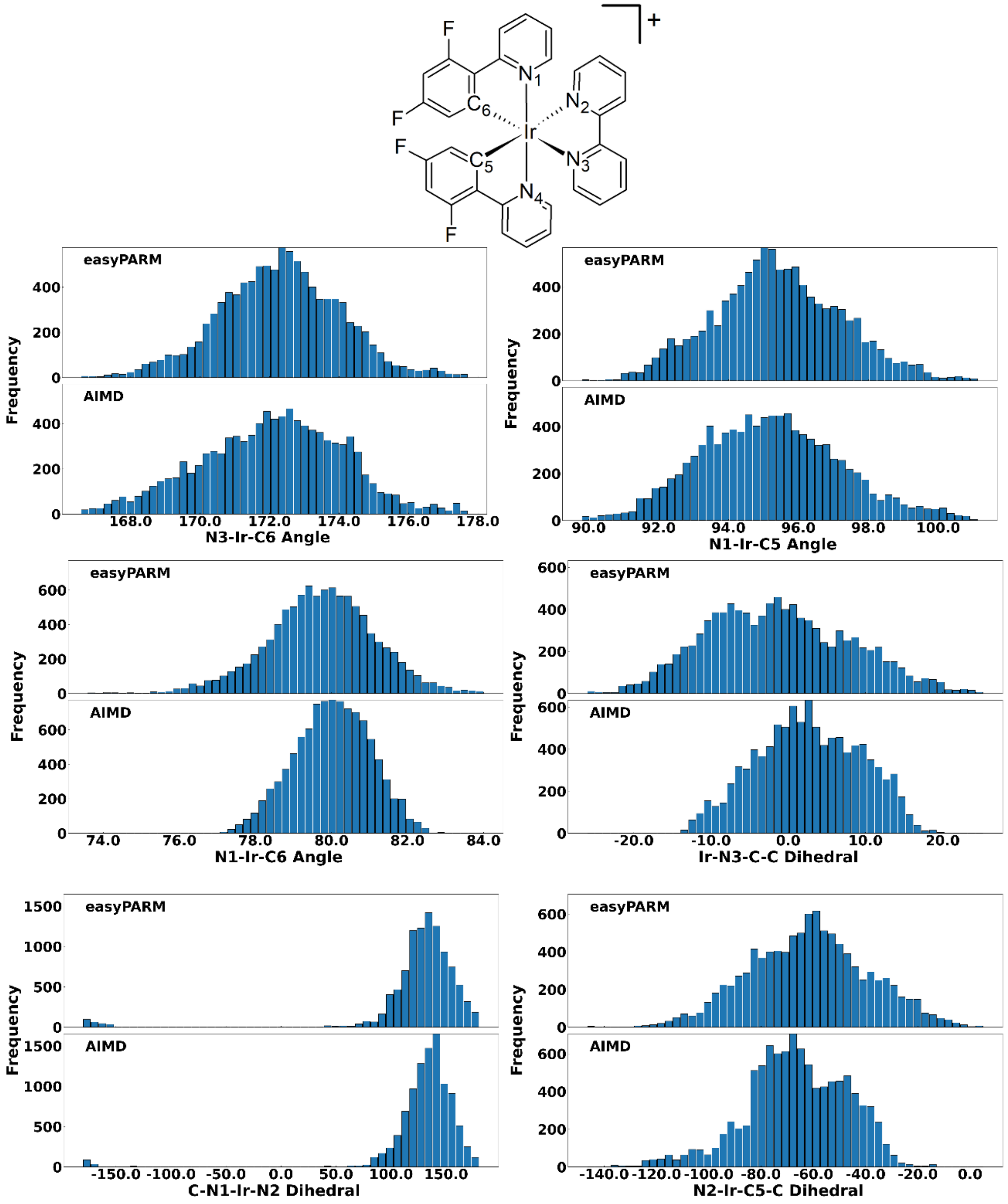

Figure S6. Histograms of selected parameters involving the metal center for Structure **2**, comparing easyPARM (MD) and *ab initio* molecular dynamics (AIMD) over 10 ps.

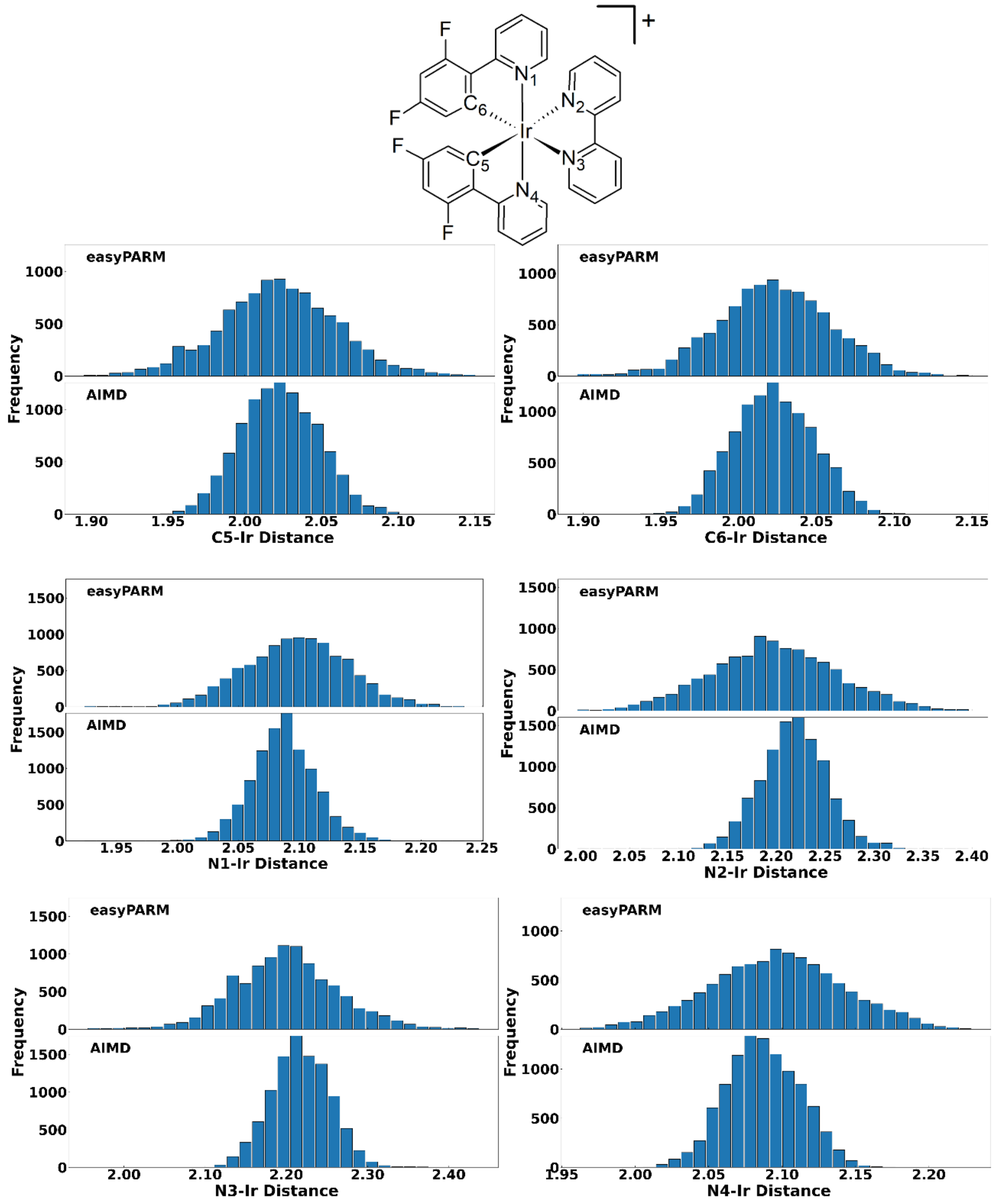

Figure S7. Histograms of selected parameters involving the metal center for Structure **2**, comparing easyPARM (MD) and *ab initio* molecular dynamics (AIMD) over 10 ps, with empirical dispersion corrections.

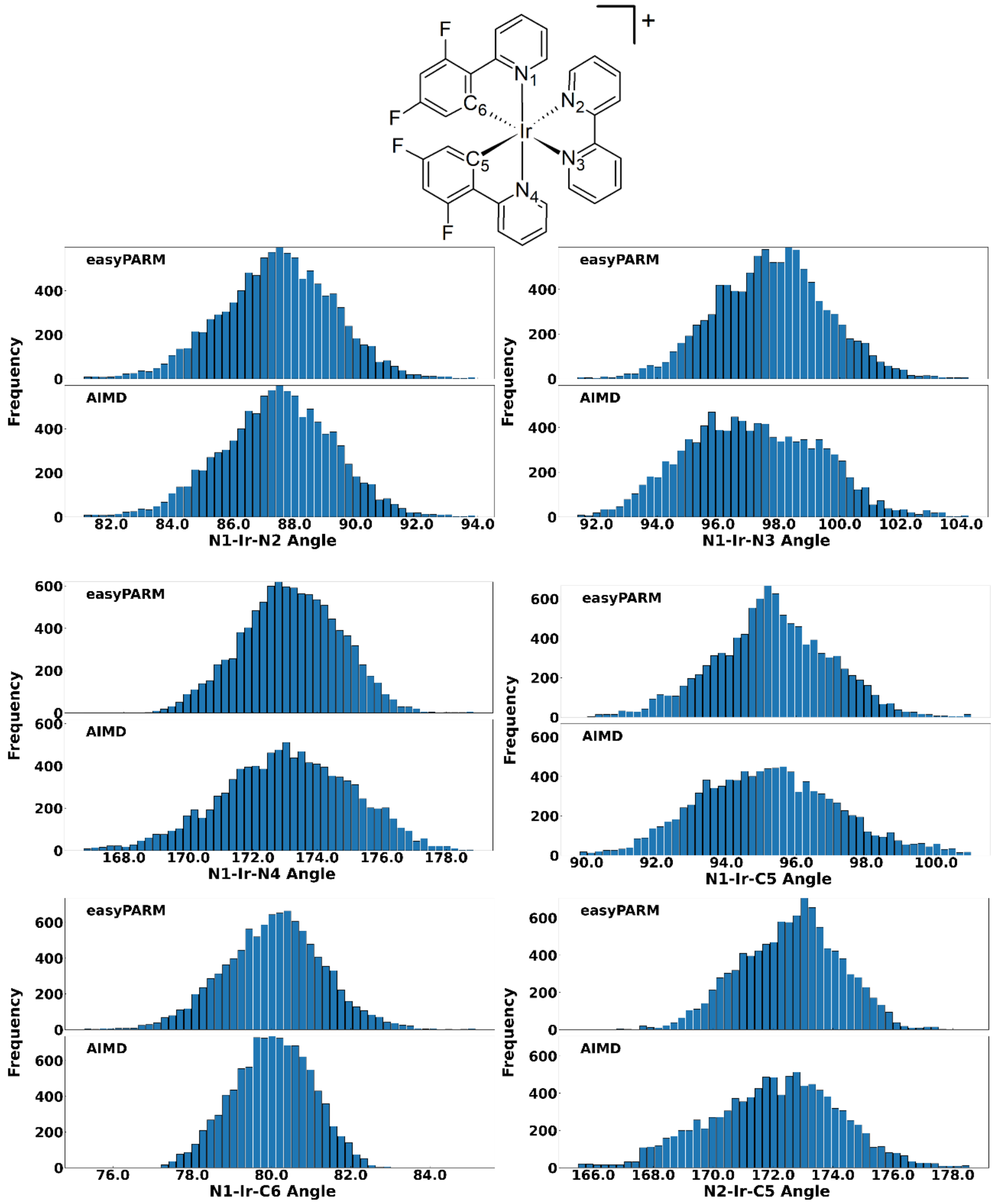

Figure S8. Histograms of selected parameters involving the metal center for Structure **2**, comparing easyPARM (MD) and *ab initio* molecular dynamics (AIMD) over 10 ps, with empirical dispersion corrections.

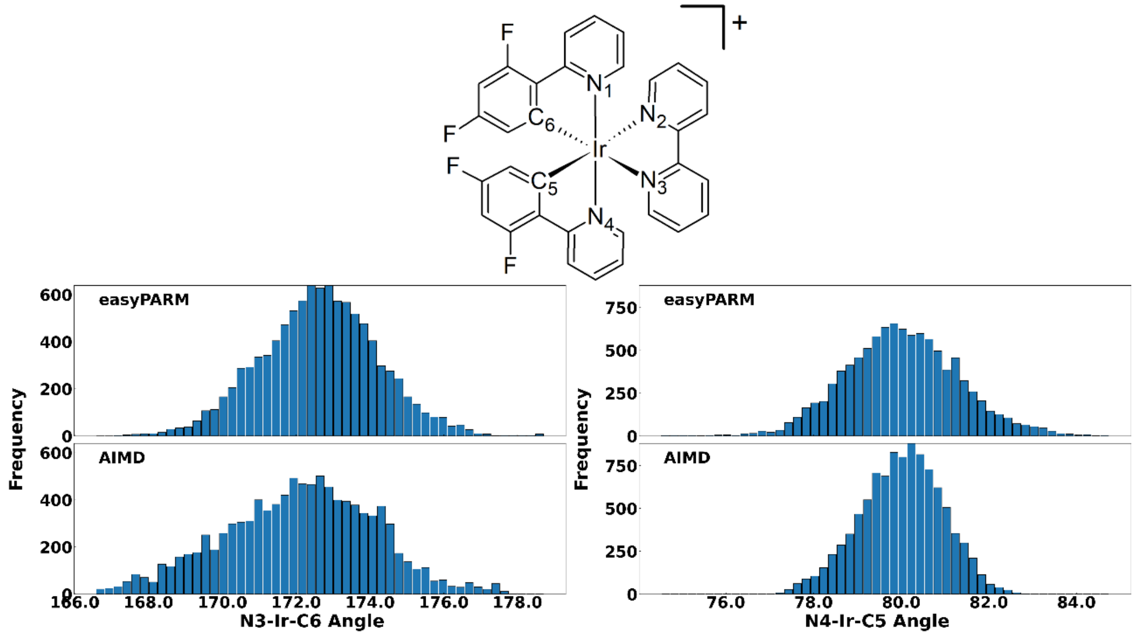

Figure S9. Histograms of selected parameters involving the metal center for Structure **2**, comparing easyPARM (MD) and *ab initio* molecular dynamics (AIMD) over 10 ps, with empirical dispersion corrections.

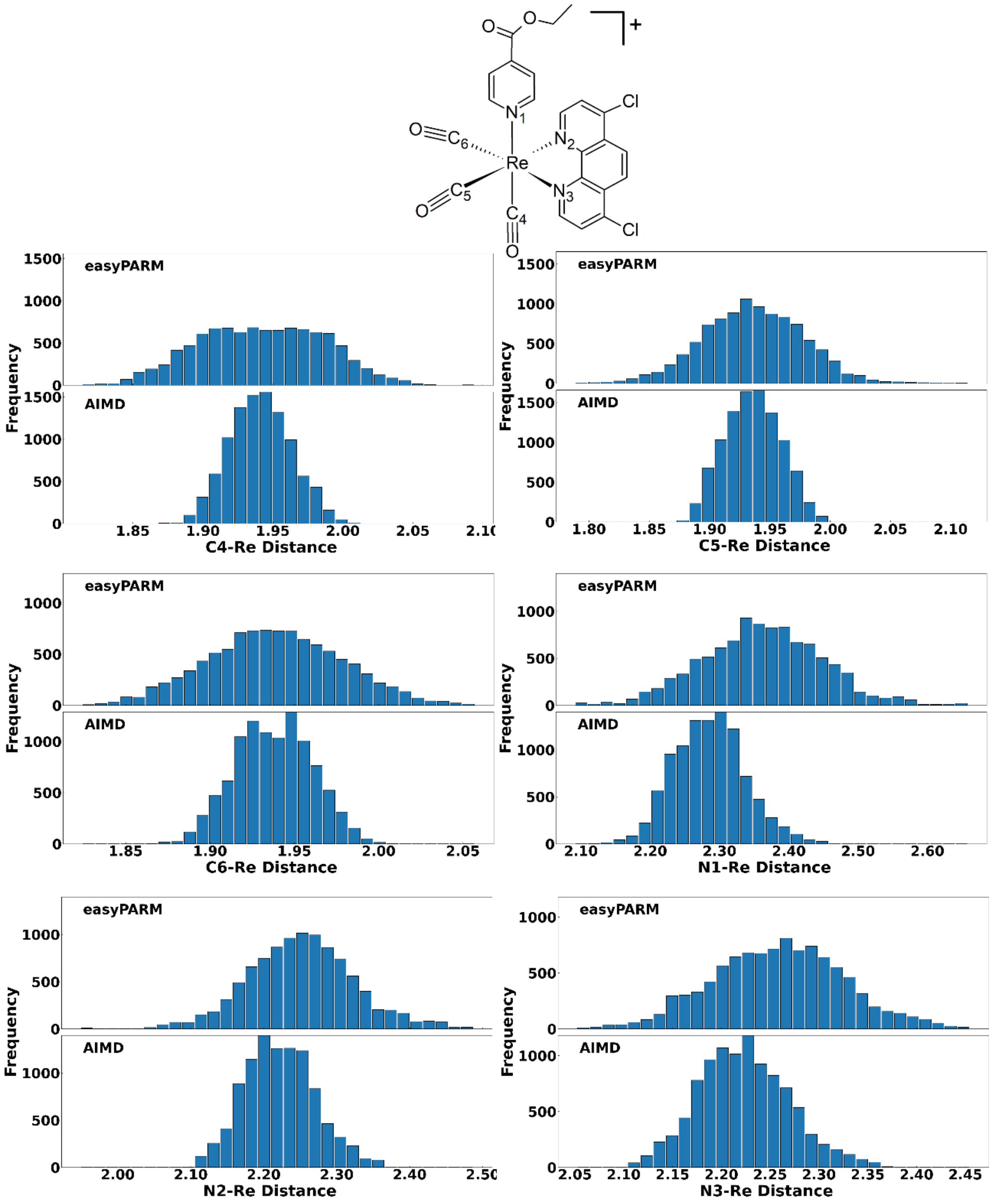

Figure S10. Histograms of selected parameters involving the metal center for Structure **3**, comparing easyPARM (MD) and *ab initio* molecular dynamics (AIMD) over 10 ps.

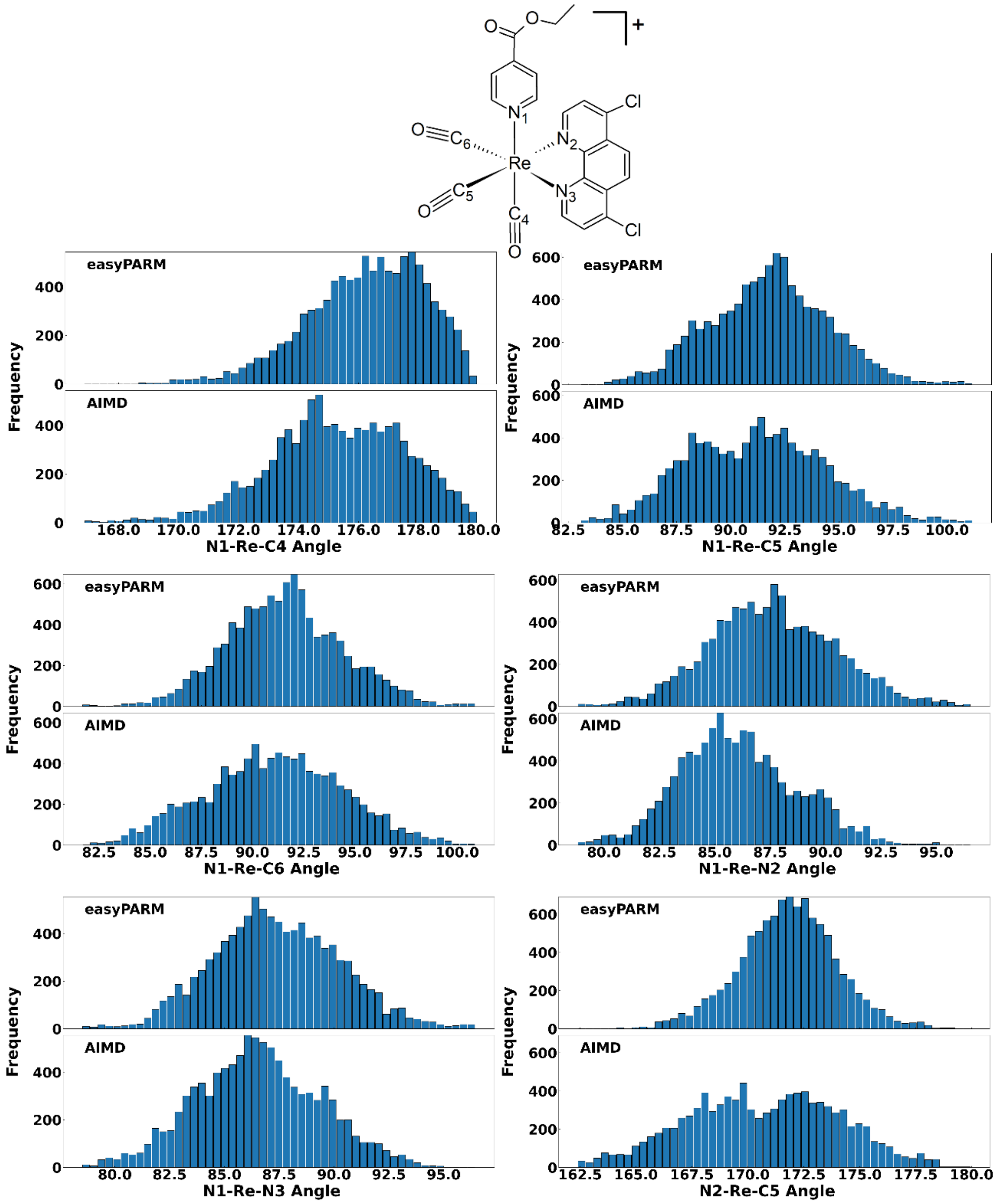

Figure S11. Histograms of selected parameters involving the metal center for Structure **3**, comparing easyPARM (MD) and *ab initio* molecular dynamics (AIMD) over 10 ps.

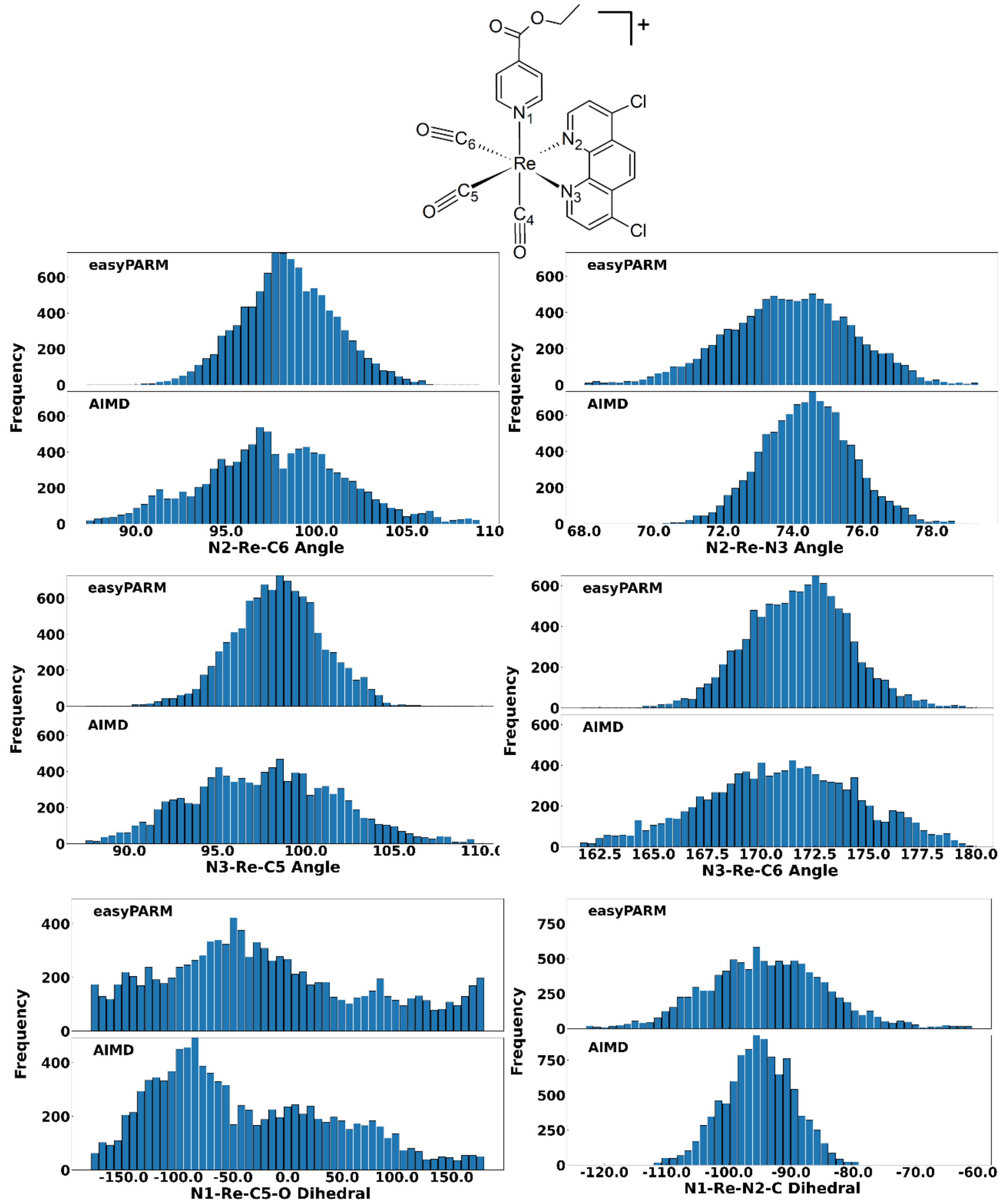

Figure S12. Histograms of selected parameters involving the metal center for Structure **3**, comparing easyPARM (MD) and *ab initio* molecular dynamics (AIMD) over 10 ps.

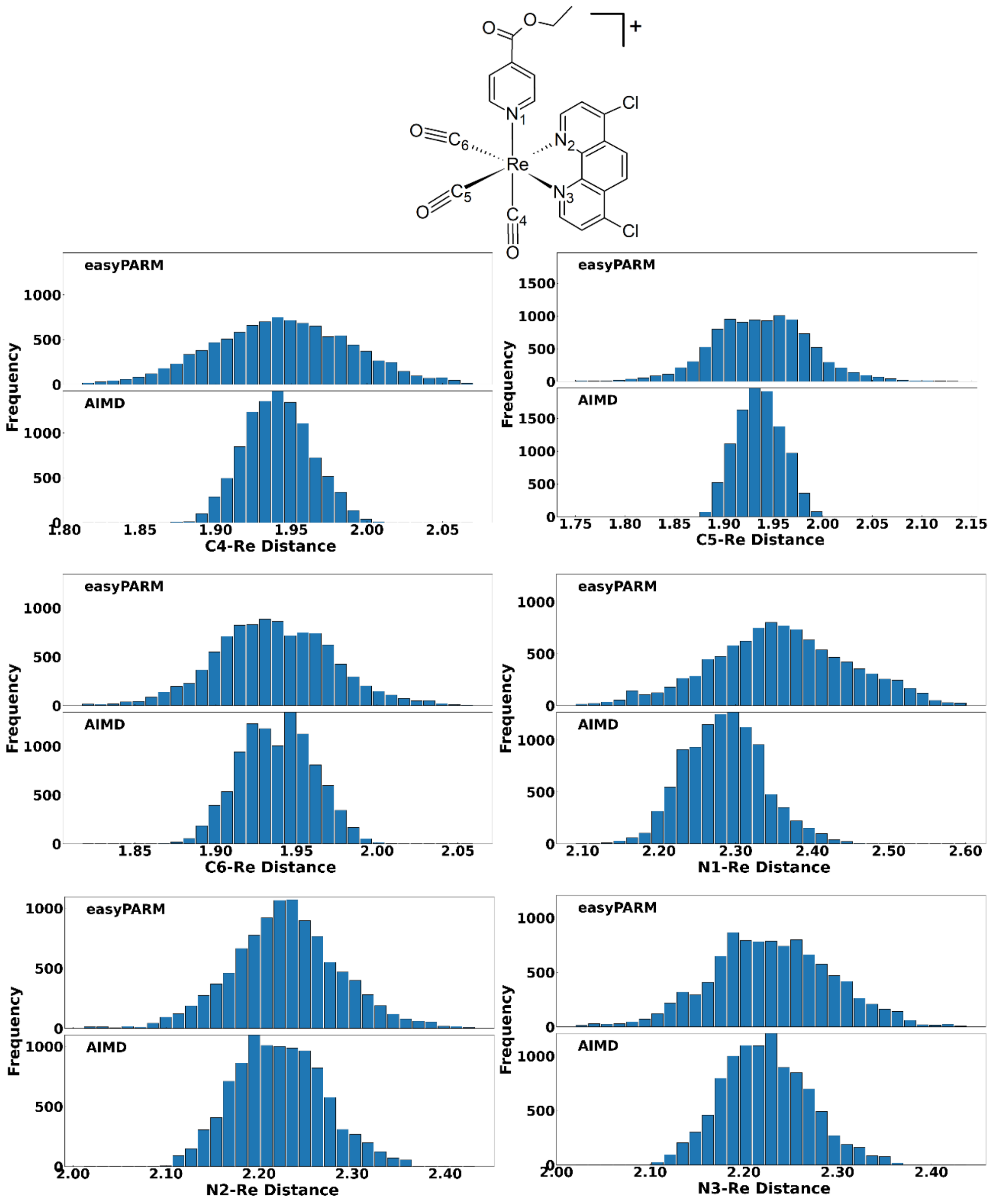

Figure S13. Histograms of selected parameters involving the metal center for Structure **3**, comparing easyPARM (MD) and *ab initio* molecular dynamics (AIMD) over 10 ps.

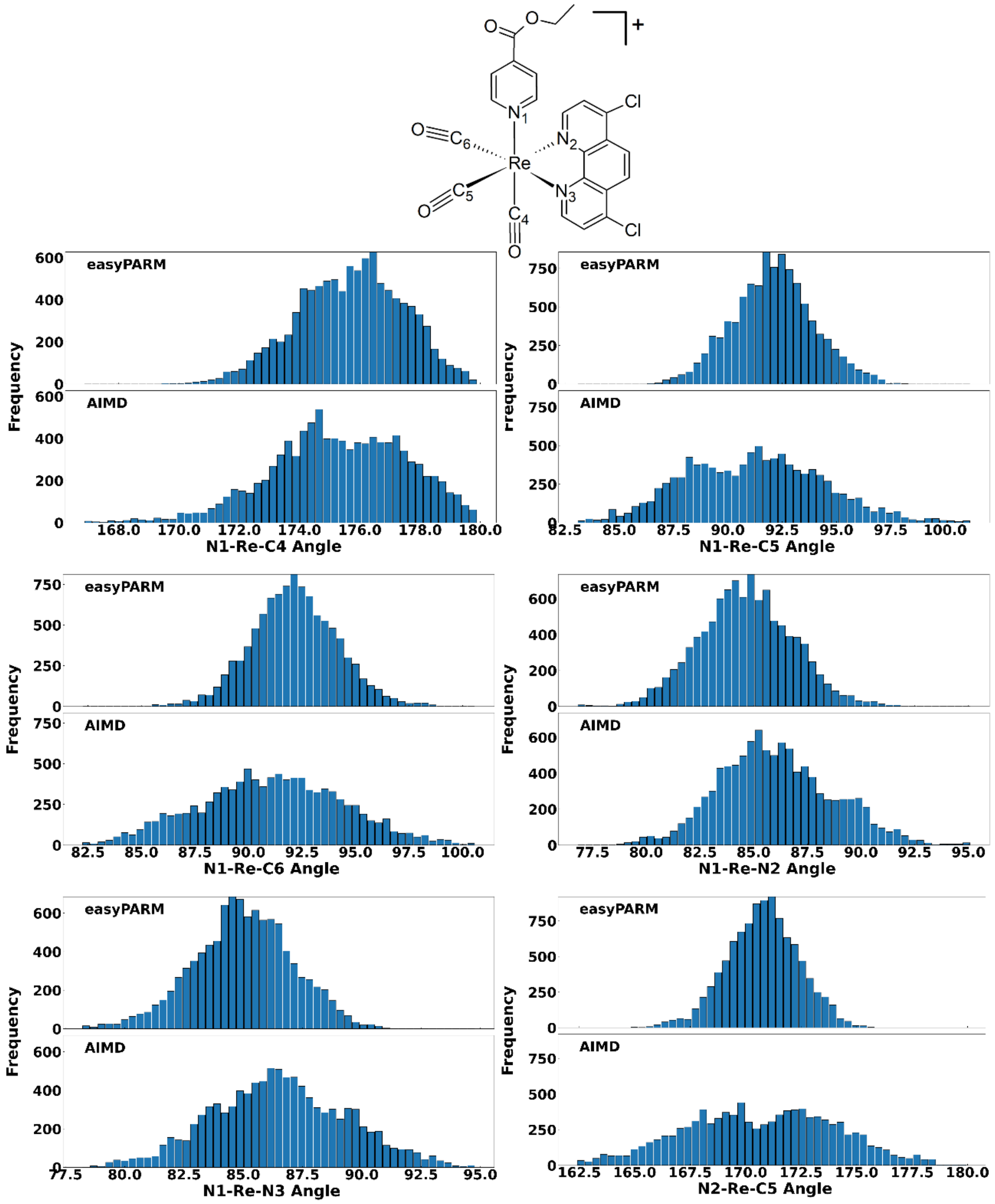

Figure S14. Histograms of selected parameters involving the metal center for Structure **3**, comparing easyPARM (MD) and *ab initio* molecular dynamics (AIMD) over 10 ps.

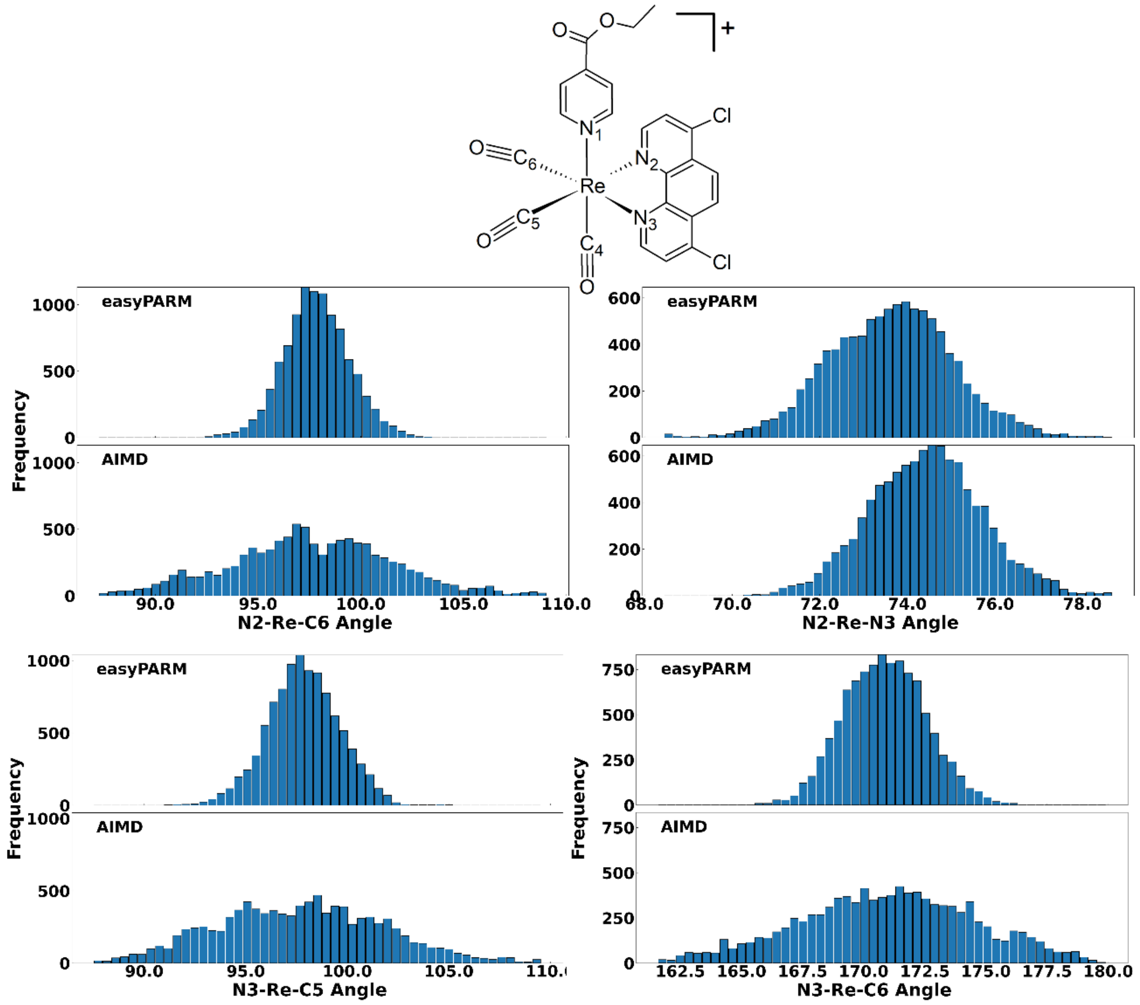

Figure S15. Histograms of selected parameters involving the metal center for Structure **3**, comparing easyPARM (MD) and *ab initio* molecular dynamics (AIMD) over 10 ps.

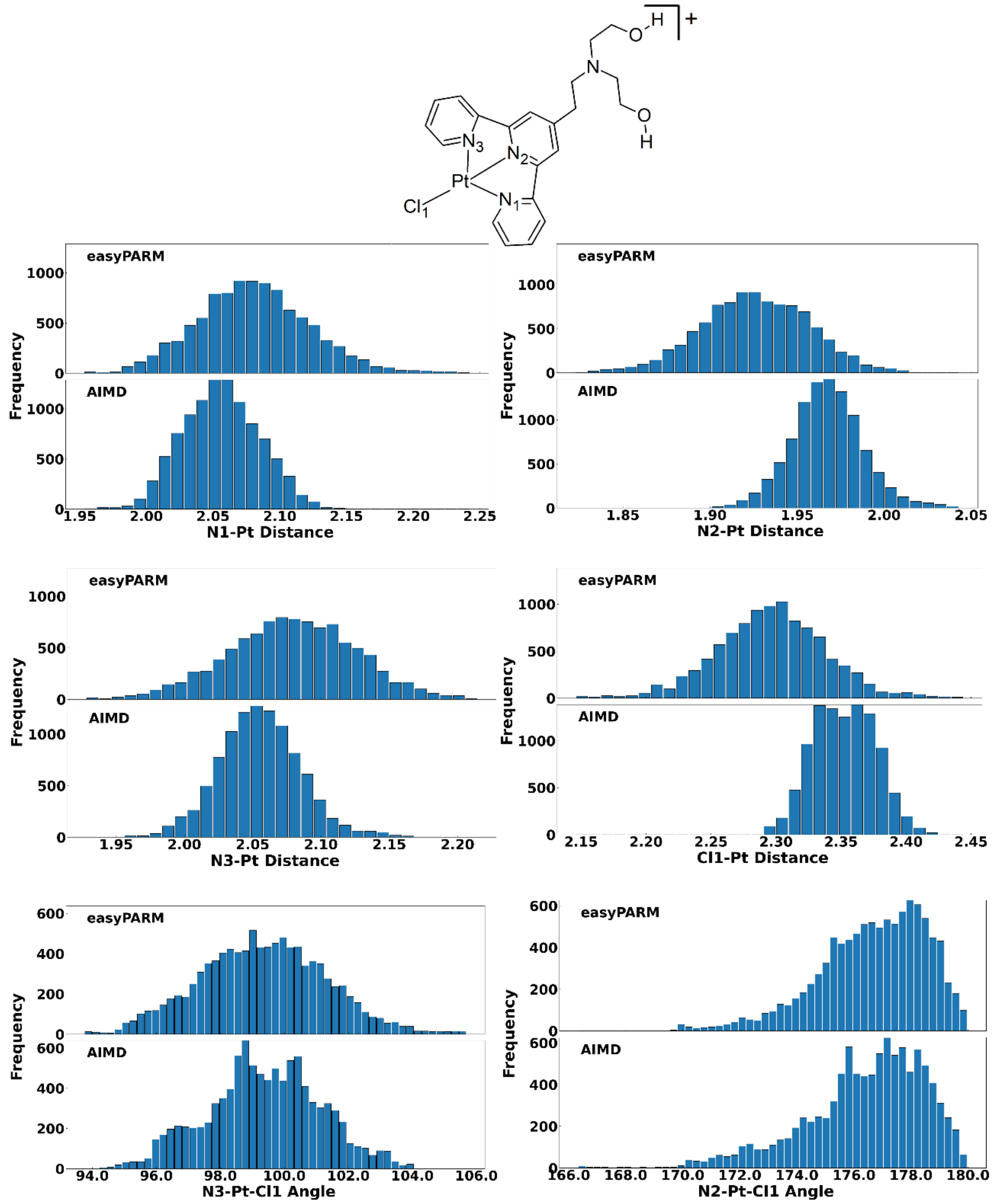

Figure S16. Histograms of selected parameters involving the metal center for Structure **4**, comparing easyPARM (MD) and *ab initio* molecular dynamics (AIMD) over 10 ps.

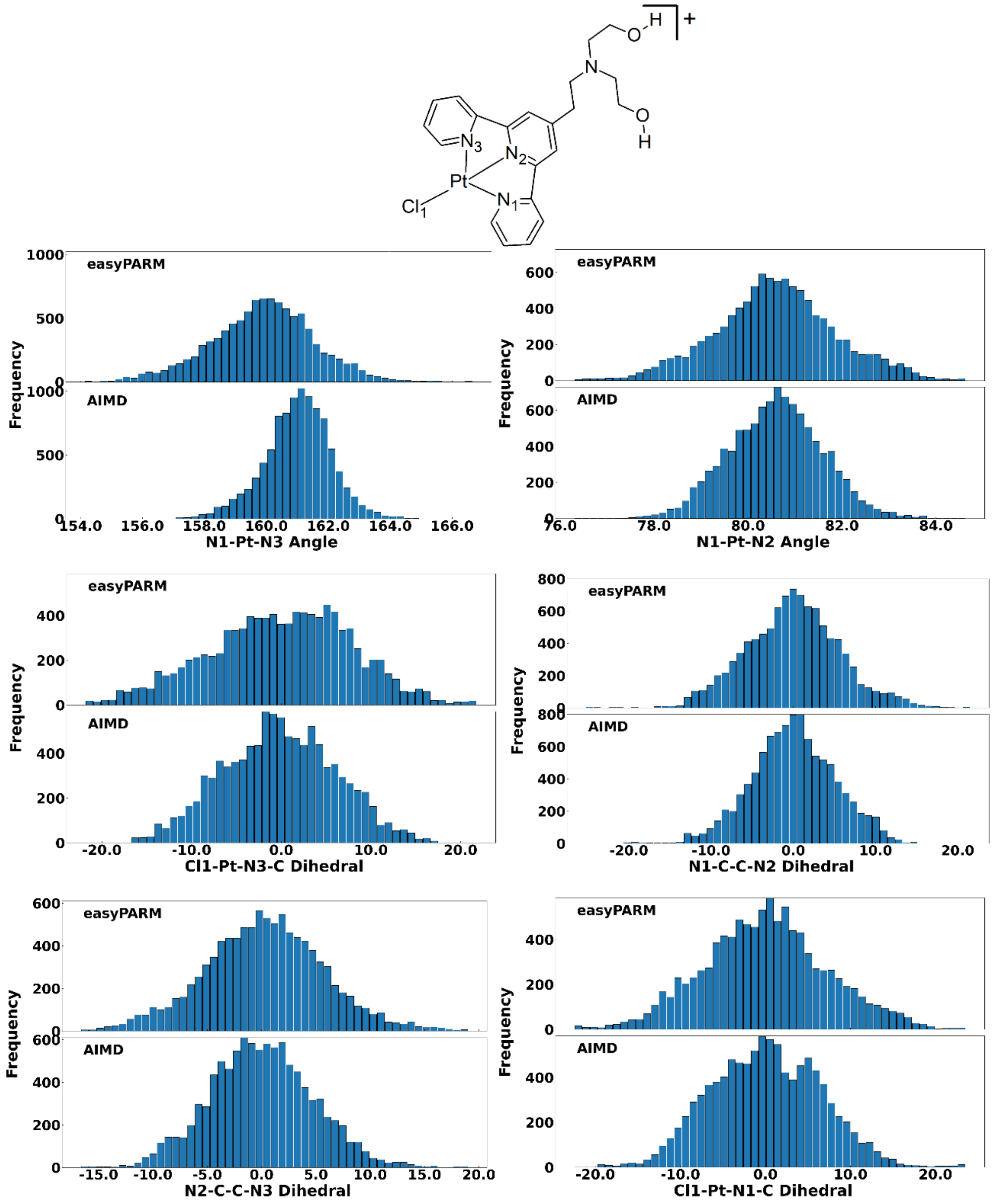

Figure S17. Histograms of selected parameters involving the metal center for Structure **4**, comparing easyPARM (MD) and *ab initio* molecular dynamics (AIMD) over 10 ps.

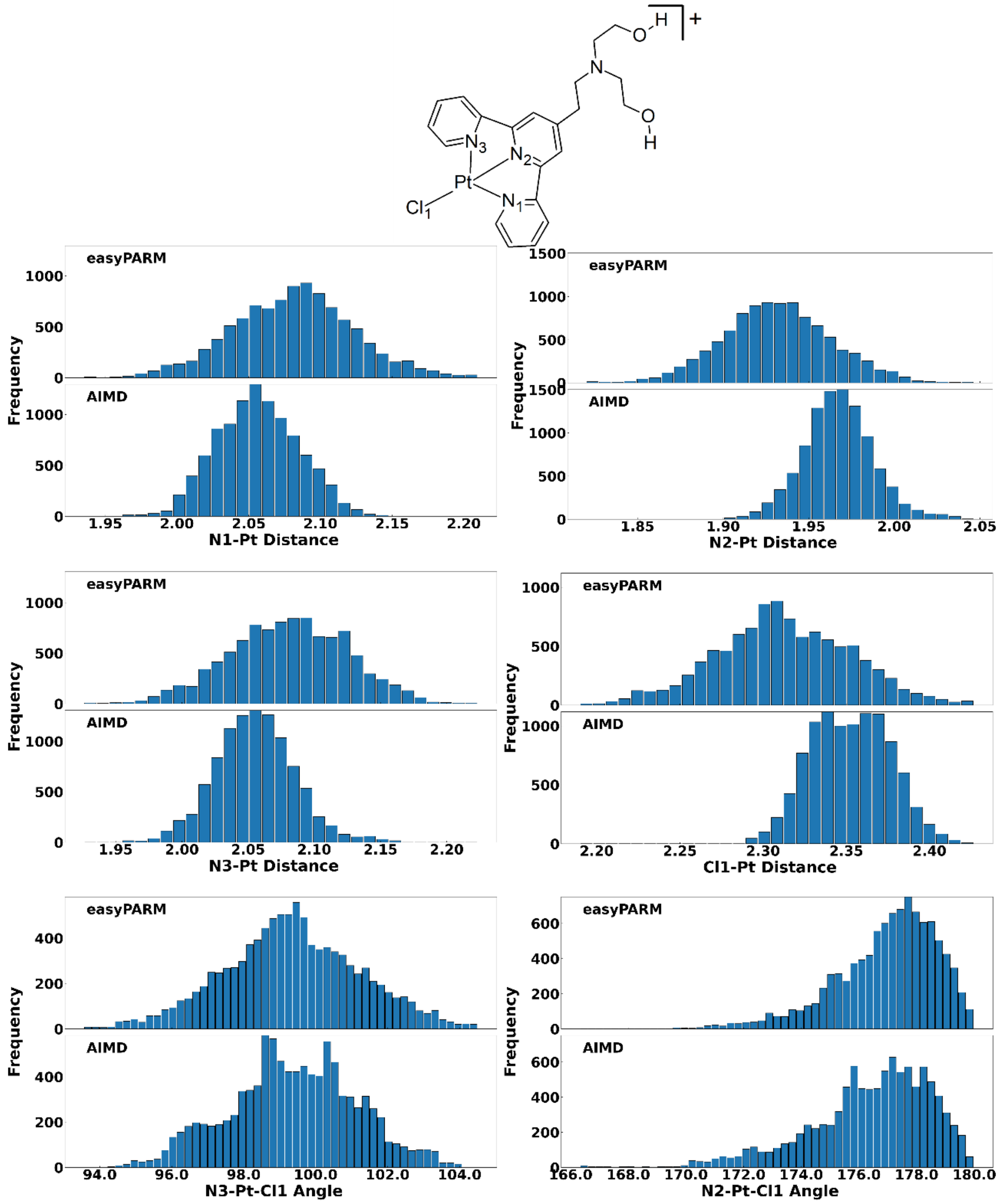

Figure S18. Histograms of selected parameters involving the metal center for Structure **4**, comparing easyPARM (MD) and *ab initio* molecular dynamics (AIMD) over 10 ps, with empirical dispersion corrections.

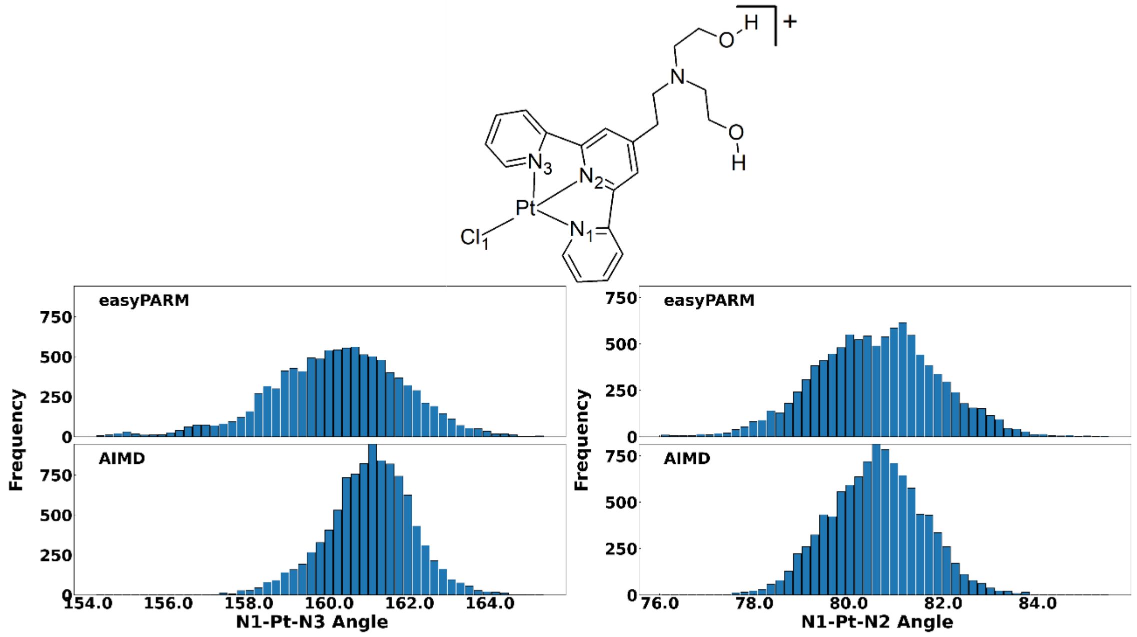

Figure S19. Histograms of selected parameters involving the metal center for Structure **4**, comparing easyPARM (MD) and *ab initio* molecular dynamics (AIMD) over 10 ps, with empirical dispersion corrections.

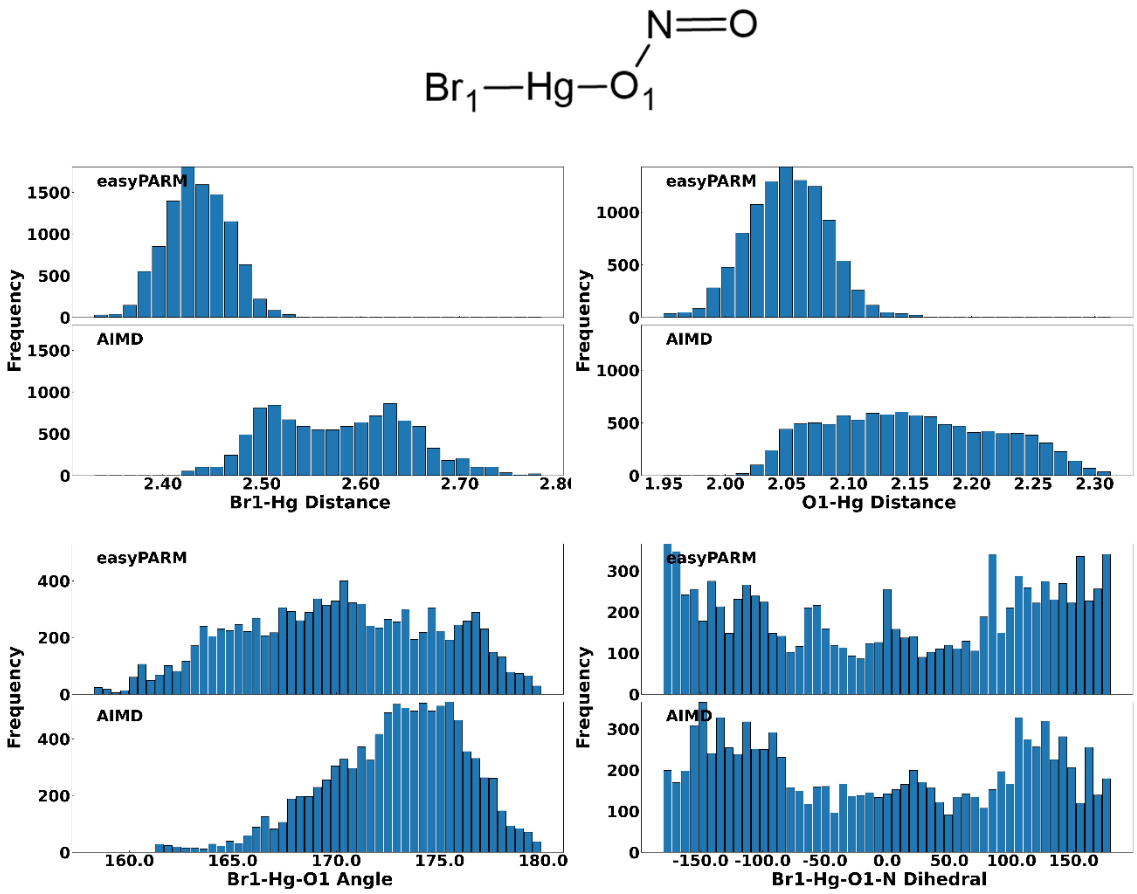

Figure S20 Histograms of selected parameters involving the metal center for Structure **5**, comparing easyPARM (MD) and *ab initio* molecular dynamics (AIMD) over 10 ps.

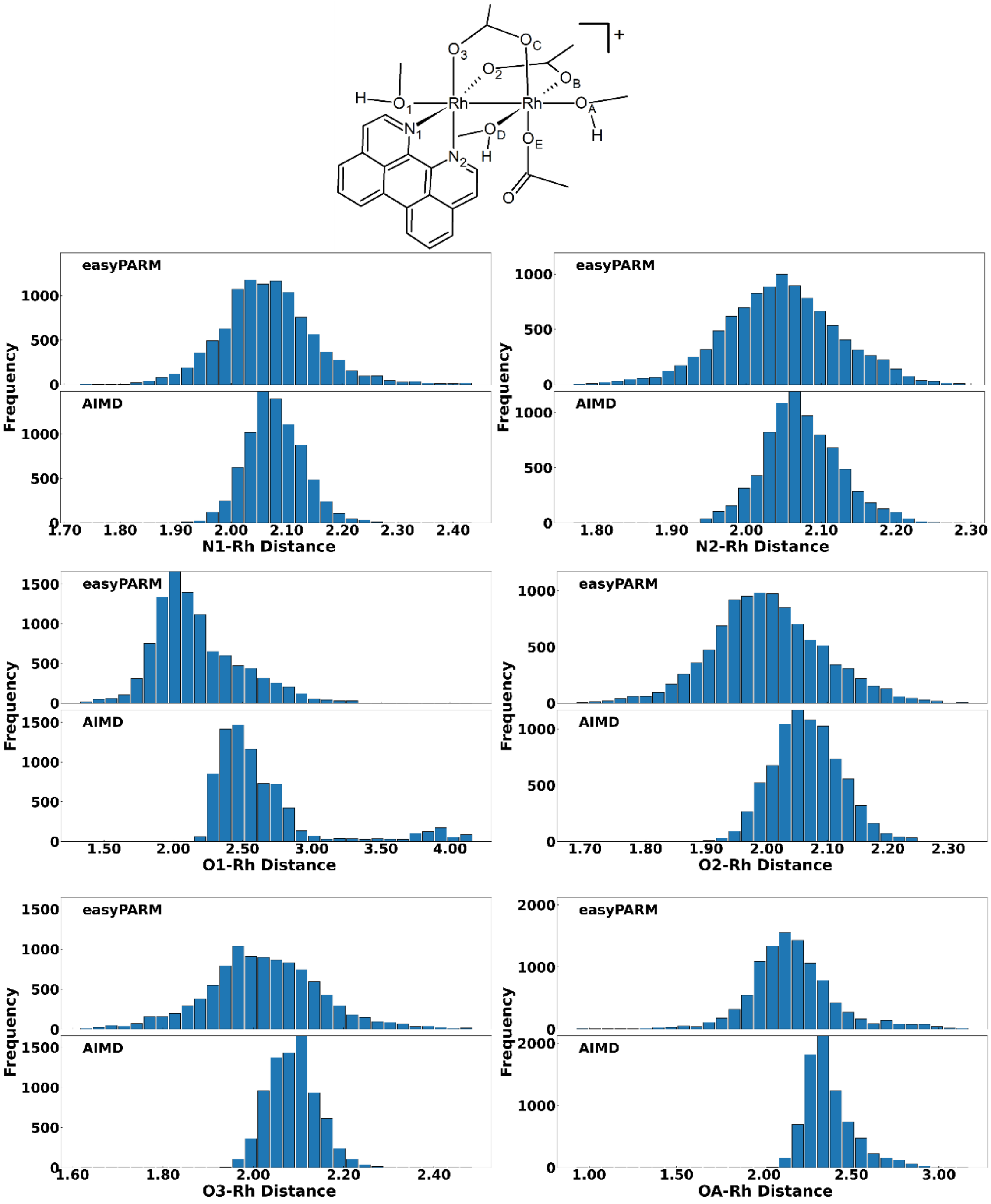

Figure S21. Histograms of selected parameters involving the metal center for Structure **6**, comparing easyPARM (MD) and quantum mechanics/molecular mechanics (QM/MM) simulations over 10 ps.

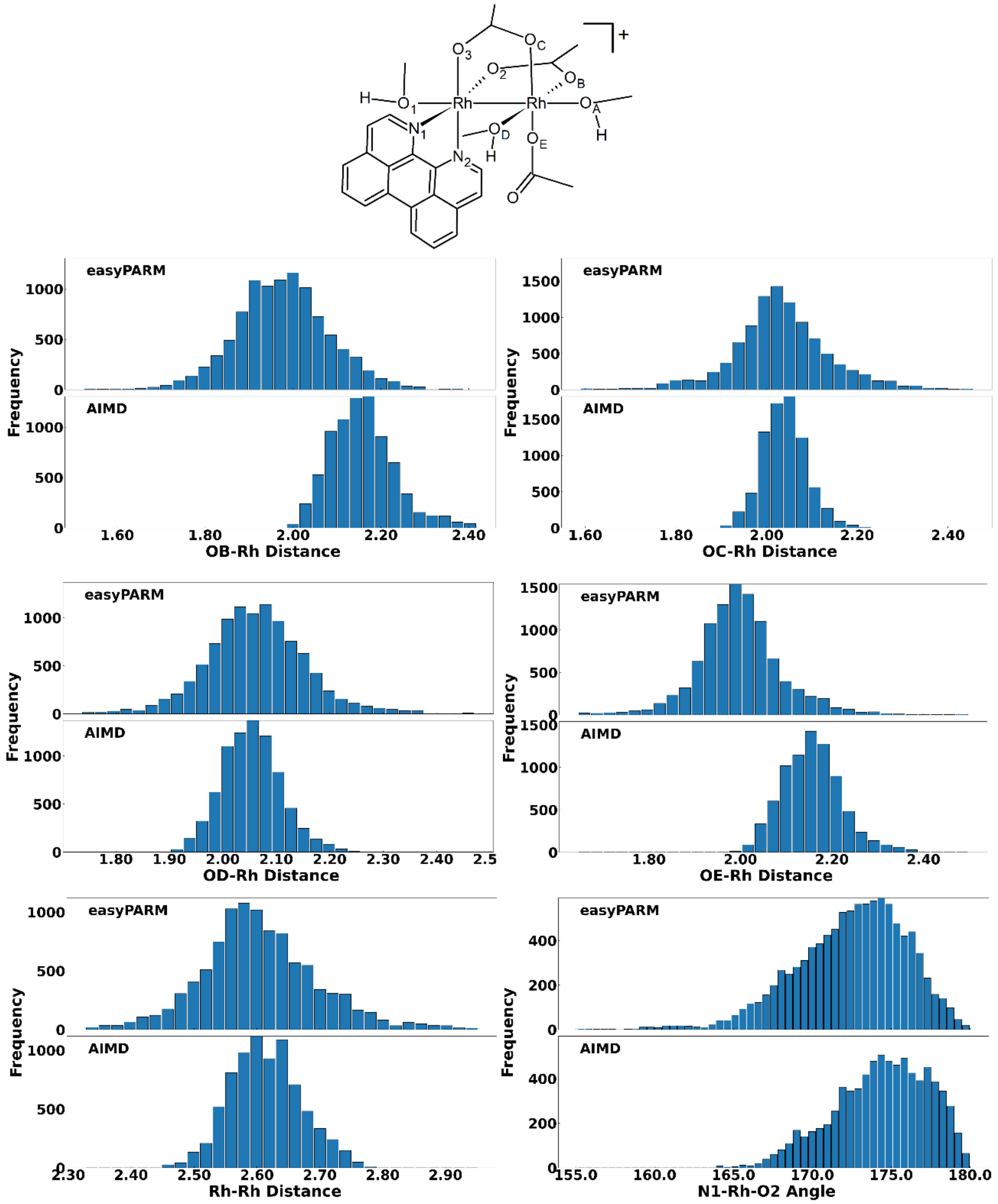

Figure S22. Histograms of selected parameters involving the metal center for Structure **6**, comparing easyPARM (MD) and quantum mechanics/molecular mechanics (QM/MM) simulations over 10 ps.

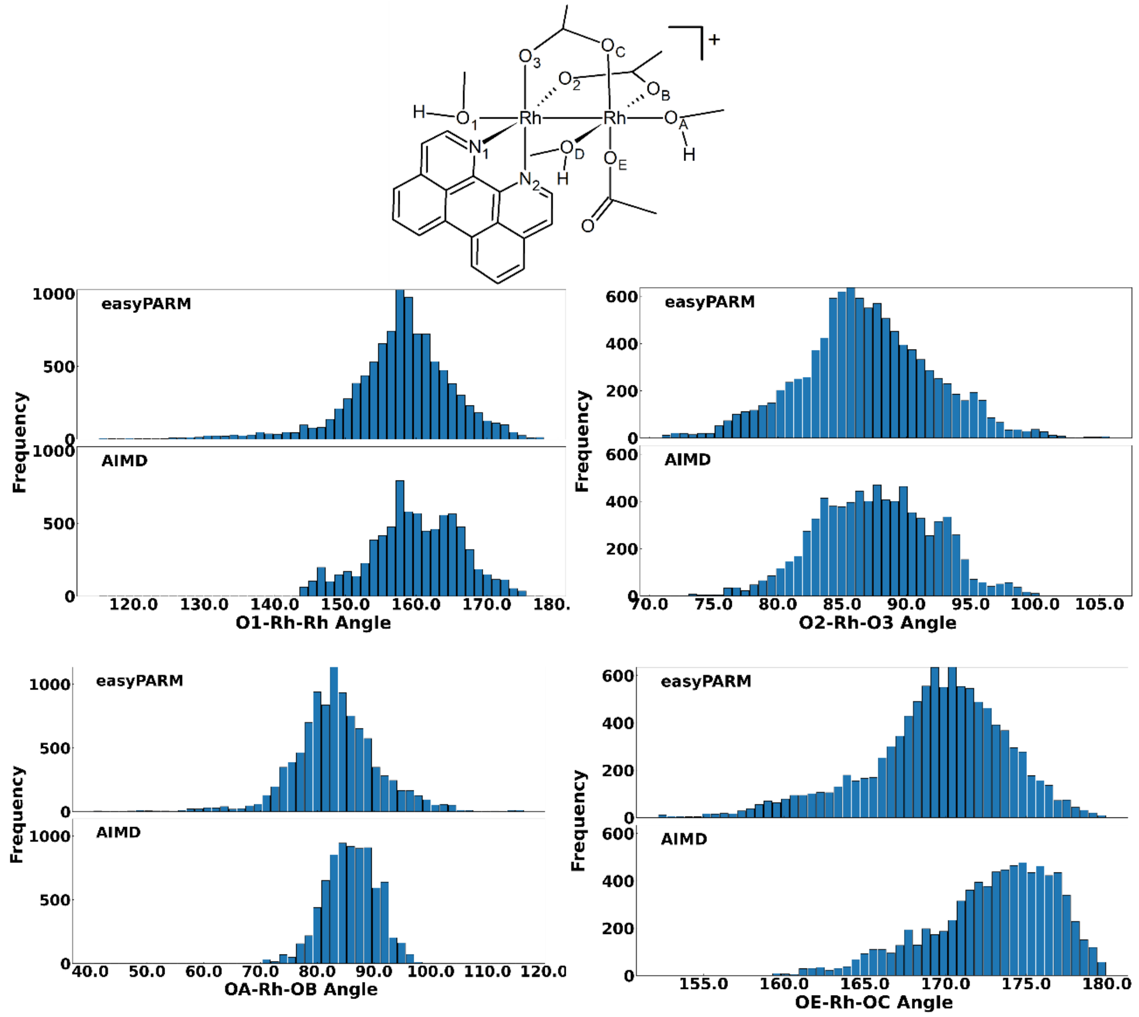

Figure S23. Histograms of selected parameters involving the metal center for Structure **6**, comparing easyPARM (MD) and quantum mechanics/molecular mechanics (QM/MM) simulations over 10 ps.

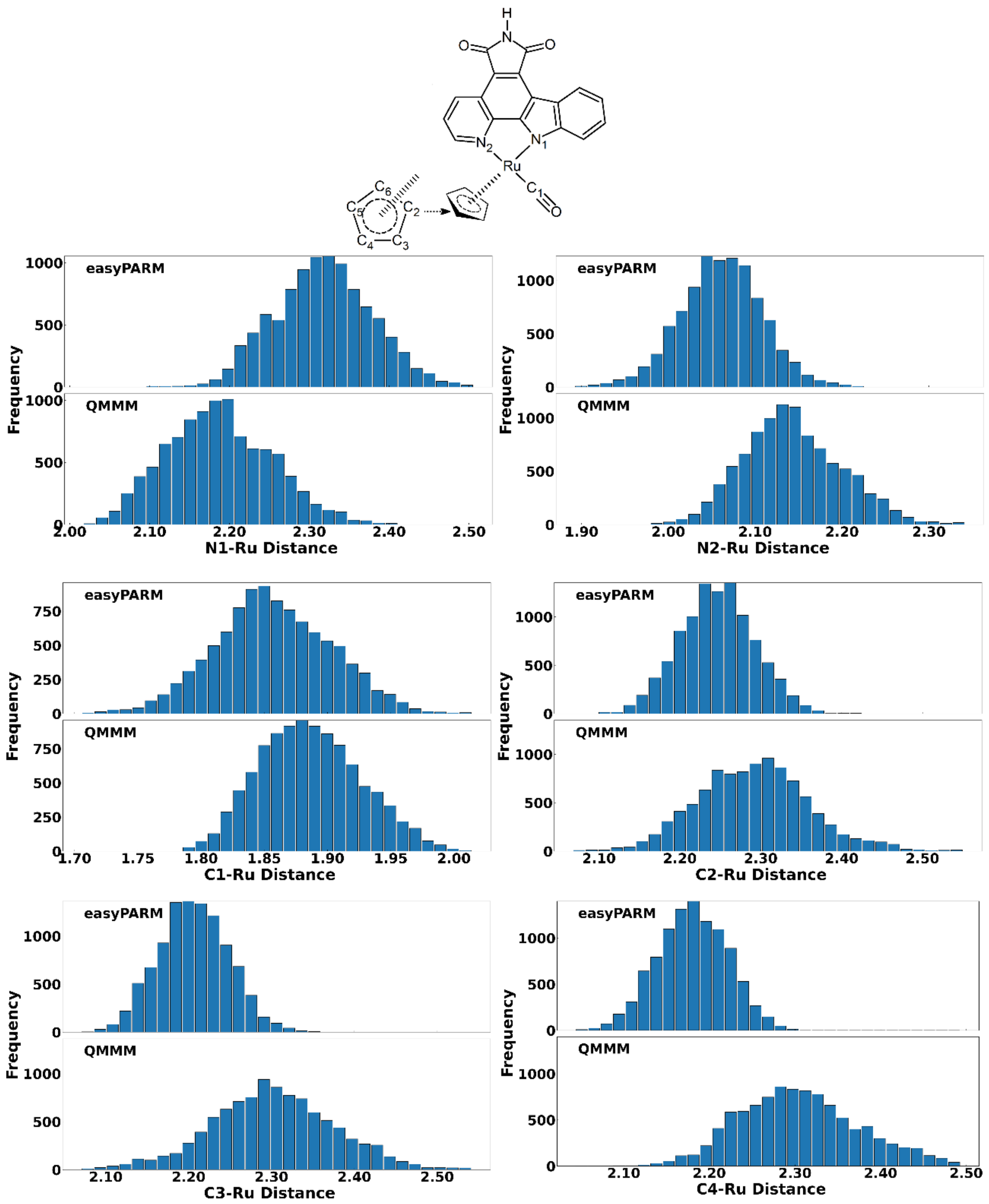

Figure S24. Histograms of selected parameters involving the metal center for Structure **7**, comparing easyPARM (MD) and quantum mechanics/molecular mechanics (QM/MM) simulations over 10 ps.

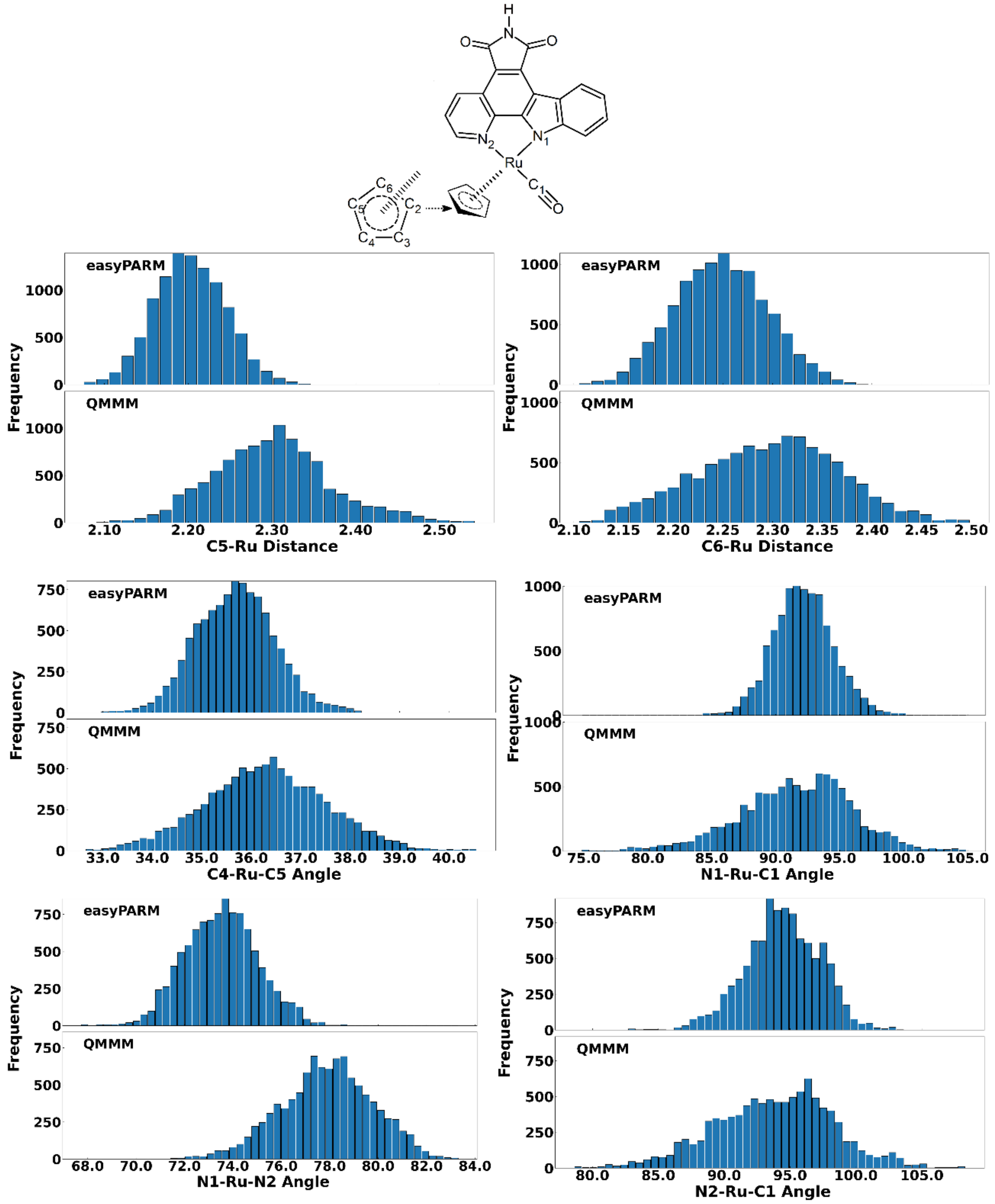

Figure S25. Histograms of selected parameters involving the metal center for Structure **7**, comparing easyPARM (MD) and quantum mechanics/molecular mechanics (QM/MM) simulations over 10 ps.

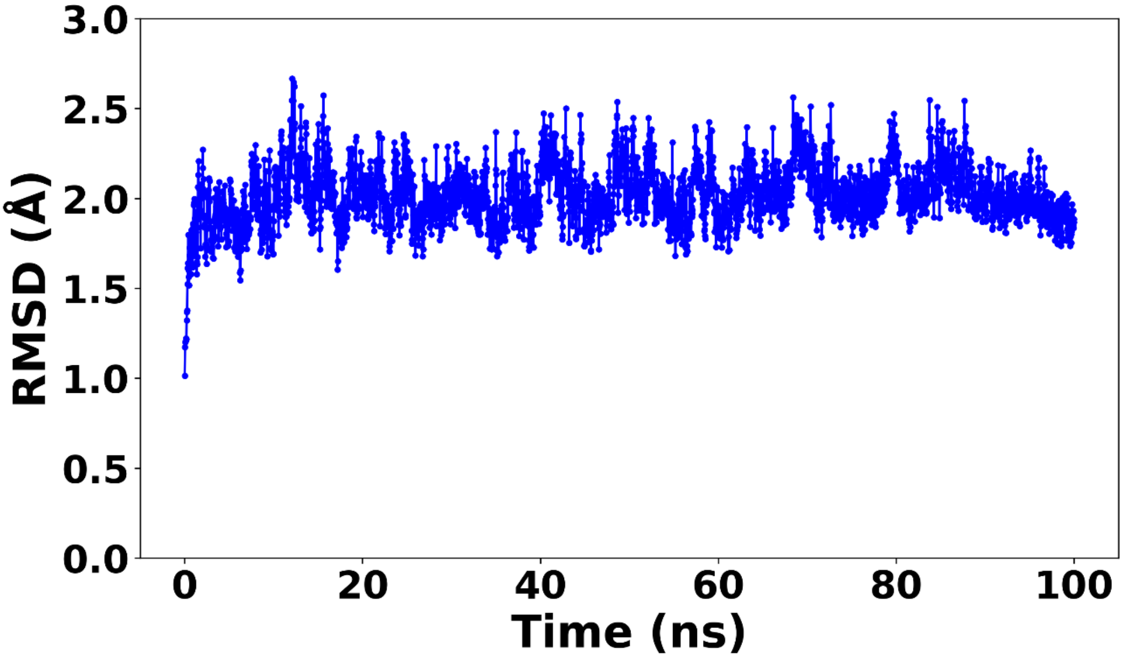

Figure S26. Root mean square deviation (RMSD) for the protein/**7** complex (PDB ID: 2BZH) simulated with easyPARM parameters for 100 ns.

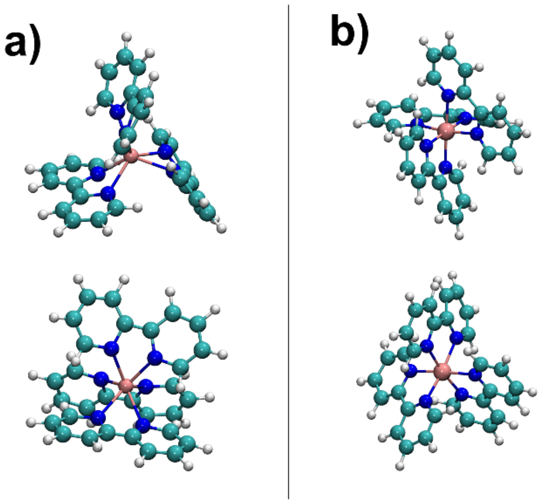

Figure S27. Last snapshot of the 10 ps simulation of complex **1** with a) all N atoms coordinating Ru have the same label, and b) every N atom coordinating the Ru center has a unique label (ULS).
